# Hydroxyl-modified chitosan nanofiber beads for sustainable boron removal and environmental applications†

**DOI:** 10.1039/d5ra00077g

**Published:** 2025-03-04

**Authors:** Ho Hong Quyen, Hoang M. Nguyen, Vu Chi Mai Tran, Phuoc-Cuong Le, Masashi Kurashina, Mikito Yasuzawa, Yuki Hiraga

**Affiliations:** a The University ofDa Nang – University of Science and Technology 54 Nguyen Luong Bang Street, Lien Chieu District Da Nang City 550000 Vietnam hhquyen@dut.udn.vn nmhoang@dut.udn.vn; b Department of Applied Chemistry, Graduate School of Science and Technology, Tokushima University 2-1 Minamijosanjima-cho, Tokushima-shi Tokushima 770-8506 Japan; c Department of Chemical and Biological Technologies, Shikoku Research Institute, Inc. 2109, Yashima-Nishimachi Takamatsu-shi Kagawa 761-0192 Japan

## Abstract

The removal of boron from wastewater is essential for protecting environmental health and supporting sustainable urbanization by preventing toxic accumulation in ecosystems. Existing adsorption technologies face challenges such as limited capacity, slow kinetics, high regeneration costs, and reduced efficiency due to adsorbent saturation. This study develops an eco-friendly adsorbent (CGCNF beads) by modifying chitosan nanofibers with d-(+)-glucono-1,5-lactone (GL) to enhance boron removal. The adsorbents were characterized by ^1^H NMR, Cosy NMR, SEM, BET, TGA, FTIR, and colloidal titration. Notably, the hydroxyl functional groups grafted onto chitosan nanofibers (49.5%) were found to be three times higher than those on chitosan flakes (16.4%). The CGCNF beads followed the Langmuir model and pseudo-second-order model with a maximum boron adsorption capacity of 6.05 mg g^−1^, surpassing commercial Amberlite IRA-743 resin (5.73 mg g^−1^). The adsorption process of CGCNF beads was much faster, reaching equilibrium in 120 minutes, compared to 720 minutes for adsorbent-based chitosan flakes. The adsorption capacity is significantly enhanced by either elevating the pH levels or introducing salts such as NaCl, KCl, CaCl_2_, or MgCl_2_. The beads showed robust regeneration, maintaining 65.1% of their adsorption capacity after 20 cycles. The developed CGCNF beads also demonstrate simultaneous high-efficiency removal of B(iii) and As(iii) ions from local wet flue gas desulfurization (FGD) wastewater at rates of 94.5% and 100%, respectively, providing a sustainable solution for wastewater contamination.

## Abstract 2

Eco-friendly beads from chitosan nanofibers enhance boron removal from wastewater, overcoming slow kinetics, limited capacity, and high regeneration costs, providing sustainable solutions for environmental protection and urban development.

## Graphical abstract

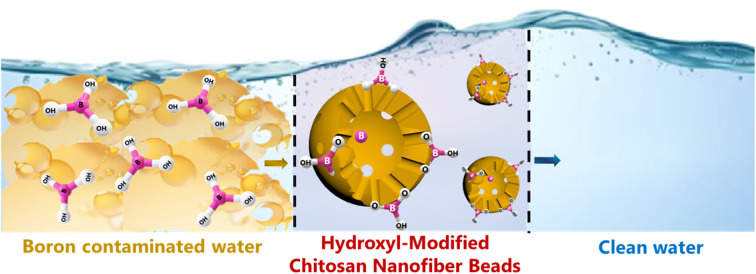

## 1 Introduction

Boron naturally occurs in compounds such as boric acid and borates found in rocks, soils, and water sources, and is released through both natural processes and human activities like mining, agriculture, and industrial emissions. While boron is essential for plant growth, excessive concentrations in irrigation water can lead to toxicity, particularly in sensitive crops, reducing agricultural yield and quality.^1^ Overexposure to boron through contaminated water and food can also pose health risks to humans, highlighting the need for regulated levels.^2^ As a result, organizations such as the WHO have set guidelines for safe boron concentrations of 2.4 mg L^−1^ for drinking water, with some countries implementing even stricter standards to protect public health.^3^

Various methods have been suggested for the removal and recovery of boron, encompassing adsorption,^4^ membrane filtration,^5^ precipitation,^6^ coagulation,^7^ electrocoagulation,^8^ and phytoremediation.^9^ Among these approaches, adsorption using an adsorbent stands out as an economical and efficient technique for eliminating dissolved boron from water and wastewater. This preference is attributed to its simplicity in operation and the limited production of secondary pollutants.^10^ The adsorption technique offers a practical and cost-effective solution, making it a favorable choice in addressing the challenge of boron removal while minimizing environmental impact. Various adsorbents, including activated carbon,^11^ fly ash,^12^ bentonite,^13^ sepiolite,^14^ illite,^15^ magnesite,^16^ kaolinite,^17^ zeolite,^18^ waste calcite,^19^ and red mud,^20^ have been investigated for boron separation from aqueous solutions. However, these materials often exhibit limitations in terms of effectiveness, stability, and regeneration. To address these shortcomings, researchers have explored alternative adsorbents aiming to enhance performance with non-biodegradable materials such as synthetic polymers,^21^ silica,^22^ or nylon fiber.^23^ Despite their effectiveness, the non-biodegradable nature of these synthetic polymer adsorbents poses environmental concerns.

Chitosan, a natural polymer abundantly available (second only to cellulose), stands out for its popularity and affordability.^24^ Notably, it is inherently biodegradable and releases non-toxic elements.^25^ Derived from the deacetylation process of chitin, a major component in the exoskeletons of crustaceans like shrimps, prawns, crabs, and the cellular walls of fungi,^26^ chitosan holds a key position in natural resources. Its composition includes hydroxyl groups (–OH) and amino groups (–NH_2_) that serve as reactive sites and primary sites for chemical modification. These features make chitosan widely applicable for various environmental applications, particularly in the removal of heavy metals and other pollutants. The combination of its abundant availability, biodegradability, and versatility in chemical modification underscores chitosan's significance as an eco-friendly and effective material for diverse applications in pollution control and remediation processes.^27^ Although chitosan owns excellent properties, the high crystallinity, low porosity and limited surface areas of its usual forms such as flakes and powder can decrease the mass transfer and the adsorption efficiency.^28^ Modifying and optimizing the functional groups of chitosan nanofibers can significantly enhance porosity and surface functionality, eliminate the step of acid dissolution, and improve adsorption efficiency.^29^ Moreover, crosslinking augments the stability, acid resistance, and reusability of the material, surpassing the performance of traditional adsorbents.

Here, an eco-friendly adsorbent, the crosslinked gluconated chitosan nanofiber (CGCNF) beads, specifically designed for highly effective boron removal from aqueous solutions is prepared. The streamlined and simplified preparation process of CGCNF beads sets it apart, requiring fewer reagents compared to conventional methods employing chitosan flakes. This distinctive combination of utilizing chitosan nanofibers and the simplified synthesis process constitutes the unique contribution and novelty of our study in the realm of boron removal adsorbents. Characterization of the resulting CGCNF beads is thoughtfully conducted using ^1^H NMR, Cosy NMR, SEM, BET, TGA, FTIR, and colloidal titration. Boron adsorption isotherms, kinetics, and potential adsorption mechanisms are explored under various conditions, including initial pH, initial boron concentration, contact time, and the presence of chloride salts including NaCl, KCl, CaCl_2_, and MgCl_2_. This work also delves into the regeneration and reusability of the CGCNF beads. Furthermore, the adsorption capacity of CGCNF beads for Se(vi), As(iii), As(v), Cr(iii), and Cr(vi), as well as the adsorption of ions in wastewater from a wet flue gas desulfurization (FGD) system in a coal-fired power plant, were investigated. This work aims to contribute to sustainable water treatment and environmental remediation practices by introducing CGCNF beads as a versatile and efficient adsorbent. The overarching vision is to address global challenges related to water quality and environmental pollution with eco-friendly materials, offering a scalable solution that extends beyond specific applications to promote a broader impact on environmental sustainability and human well-being.

## 2 Experimental

### 2.1. Materials and reagents

Chitosan nanofibers 2% (wt%, 20–50 nm average diameter) were sourced from Sugino Machine Ltd., Japan. Chitosan flakes, GL (C_6_H_10_O_6_), and ethylene glycol diglycidyl ether (EGDE) (purity > 99.8%) were supplied by Tokyo Chemical Industry Co., Ltd, Japan. Boron standard solution (1000 mg L^−1^) and boric acid (H_3_BO_3_, purity > 99.5%) for preparing boron stock solutions were purchased from Kanto Chemical Co., Inc., Japan. Sodium chloride (NaCl, purity > 99%), potassium chloride (KCl, purity > 99.5%), calcium chloride (CaCl_2_, purity > 95%), hydrochloric acid (HCl, purity: 35–37%) and sodium hydroxide (NaOH, purity > 99.5%), and magnesium chloride hexahydrate (MgCl_2_·6H_2_O, purity > 99%), were supplied by Kanto Chemical Co., Inc., Japan. The dialysis membrane (14 000 molecular weight cut-off) was provided by Thermo Fisher Scientific Inc., USA. Toluidine blue indicator solution (C_15_H_16_CIN_3_S, 0.5%, pH = 7.0) and N/400 potassium polyvinyl sulfate solution were purchased from FUJIFILM Wako Pure Chemical Corporation, Japan. Commercial resin Amberlite IRA-743 (particle size 500–700 μm, water retention capacity: 48–54%) was purchased from Dow Inc., USA. These chemicals and reagents were used directly without further purification. Mili-Q water (18.25 MΩ cm) system purchased from Direct-Q UV3, Merck Millipore, USA was used throughout all synthesis, adsorption, and characterization processes.

### 2.2. Preparation of adsorbents

#### 2.2.1. Preparation of crosslinked gluconated chitosan (CGC) particles

Gluconated chitosan (GC) particles using chitosan flakes were synthesized according to our previous work with some modifications.^30^ Accordingly, the mixture of 10 g of chitosan flakes and 1000 mL of acetic acid solution 1% (*v*/*v*) was stirred at room temperature. GL was added to the chitosan solution with the mole ratio of chitosan : GL (1 : 5). The mixture was refluxed and stirred at 115 °C for 24 h. The mixture was left to cool to room temperature, and then 1 M NaOH solution was added to form the particles. The mixture of particles and NaOH solution was centrifuged. The particles were added to acetone and then collected by filtration. The impurities of the GC particles were removed by dialysis against Mili-Q water using dialysis membrane. The GC particles were crosslinked in Mili-Q water with a 0.025 M EGDE solution at 50 °C for 6 h. After the reaction was completed, the mixture was cooled to room temperature, and the CGC particles were washed thoroughly with Mili-Q water. The CGC particles were finally dried in a freeze-dryer and crushed for homogeneity.

#### 2.2.2. Preparation of CGCNF beads

**Fig. 1:**
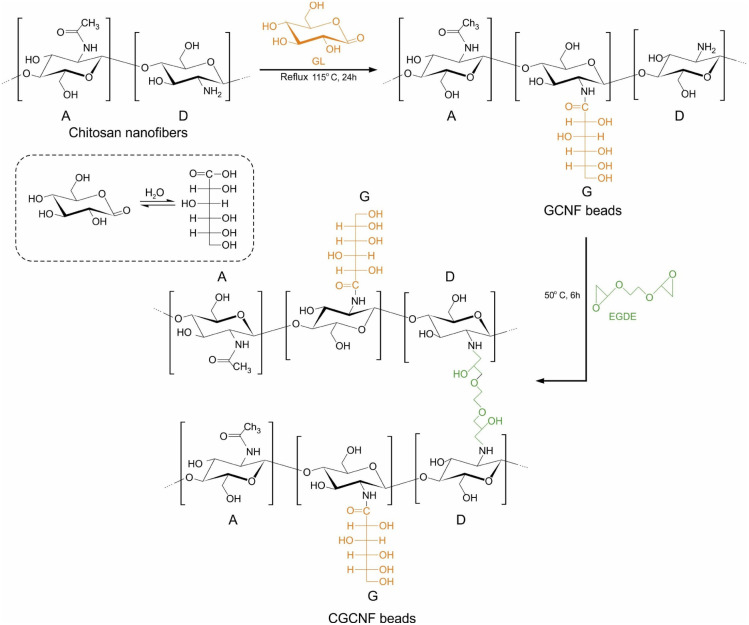
Preparation procedure of the CGCNF beads (A: acetylated unit, D: deacetylated unit, G: gluconated unit).

Gluconated chitosan nanofiber (GCNF) beads were synthesized by blending a 2% (*v*/*v*) solution of chitosan nanofibers with GL in a molar ratio of 1 : 5. The mixture underwent continuous reflux and stirring at 115 °C for 24 h to achieve a homogeneous solution. Following cooling to room temperature, dialysis against Mili-Q water using a dialysis membrane was employed to remove residual unreacted GL for 5 days. The purified solution was concentrated to form a gel through rotary evaporation at 35 °C. The gel was then dropwise introduced into a 0.1 M NaOH solution, resulting in the immediate formation of beads. Subsequently, these beads were suspended in a 0.1 M NaOH solution for one day and thoroughly washed with Mili-Q water until reaching neutral pH. The GCNF beads were immersed in a 0.025 M EGDE solution, and this mixture underwent mechanical stirring at 50 °C for 6 h to facilitate crosslinking. Finally, the resulting CGCNF beads were filtered, extensively washed with Mili-Q water, dried in a freeze dryer for 12 h, and stored in a desiccator. The pictures of CGCNF beads before and after freeze-drying are shown in Fig. S1.† The synthesis process of CGCNF beads is presented in Fig. 1.

### 2.3. Characterization

#### 2.3.1. Proton nuclear magnetic resonance (^1^H NMR) and Cosy NMR

In this work, chitosan nanofibers and GCNF beads were characterized by using ^1^H NMR (Proton Nuclear Magnetic Resonance). ^1^H NMR was selected since it provides a unique insight into the molecular structure of chitosan-based particles.^31^ It allows precise identification and quantification of individual protons in the chitosan polymer, offering a comprehensive understanding of its composition, branching, and degree of acetylation. This capability makes ^1^H NMR particularly valuable for elucidating the molecular intricacies of chitosan-based particles, complementing the information obtained from other analytical techniques. In this study, chitosan nanofibers and GCNF beads were dissolved in trifluoroacetic acid (CF_3_COOH) 2% (*v*/*v*) solution with the solvent of deuterium oxide (D_2_O) while GL was dissolved in D_2_O. ^1^H NMR data and ^1^H–^1^H Cosy NMR data were recorded by JNM-ECZ400S, Jeol, Japan which was operated at 400 MHz.

#### 2.3.2. Colloidal titration

The determination of the degree of deacetylated units (DD%) of chitosan flakes, chitosan nanofibers, GC particles and GCNF beads was performed by colloidal titration. In this experiment, 0.1 g of material, 8.6 mL of 3 M acetic acid solution and Mili-Q water were added fully into 200 mL volumetric flask. The mixture was titrated by N/400 PVSK using toluidine blue as the indicator. The titration process finished when the colour of the mixture transformed from blue to purple-pink (Fig. S2†). The titration study was carried out in quintuplicate.

#### 2.3.3. Scanning electron microscope (SEM)

FE-SEM model Hitachi S-4700, Japan was examined to observe the morphological structure of chitosan nanofibers, CGC particles, and CGCNF beads after freeze-drying.

#### 2.3.4. BET

The specific surface areas of CGCNF beads and CGC particles were determined by using ASAP 2020, Micromeritics, USA. The samples were degassed at 70 °C for 6 h before the measurements. The Brunauer–Emmett–Teller (BET) method was used to calculate the specific surface areas.

#### 2.3.5. TGA

Thermogravimetric analysis (TGA) of the prepared samples was conducted using a computer-programmed TGA/DSC-1 instrument (STA 6000, PerkinElmer, USA). Each analysis utilized 5 mg samples placed in alumina crucibles. The samples were heated from 35 to 820 °C at a rate of 10 °C min^−1^ under an air purge with a flow rate of 40 mL min^−1^.

#### 2.3.6. Fourier transform infrared spectra (FTIR)

All samples were initially finely ground using a mortar and pestle. 1.0–2.0 mg amount of each sample was then blended with 200 mg of potassium bromide (KBr), which had been thoroughly dried in microfuge tubes *via* lyophilization. The mixtures underwent further drying for 2 h in the same microfuge tubes. Subsequently, the KBr-based mixtures were compressed into thin discs under a pressure of (5–10) × 10^7^ Pa. ATR measurements were conducted with a FTIR spectrometer (VERTEX 70). The pellets were scanned at a resolution of 4 cm^−1^, performing 100 scans within the spectral range of 4000–500 cm^−1^ at ambient temperature. Dry air was continuously flowed through the sample compartment to avoid interference from water vapor. Band positions were determined based on the center of mass, with spectra from the same experimental groups being baseline corrected, normalized, and averaged.

#### 2.3.7. Stability of GC particles, CGC particles, GCNF beads and CGCNF beads

The stability of adsorbents is a crucial key for the regeneration and practical operation. In this experiment, 0.2 g of each material was immersed in 20 mL of different solutions including Mili-Q water, 0.1 M NaOH, 6 M NaOH, 0.1 M HCl and 6 M HCl for 1 month and observed the adsorbents.

### 2.4. Batch adsorption experiments

#### 2.4.1. Effect of different pH values

A total of 0.8 g of CGCNF beads were introduced into a 20 mL solution containing 400 mg L^−1^ of boric acid, with pH values ranging from 2.03 to 12.03. Initial pH adjustments were made using 0.1 M HCl and 0.1 M NaOH solutions, monitored by a pH meter (F-52, Horiba, Japan). The samples were then subjected to shaking in a water bath at 25 °C for 24 h and subsequently filtered through Whatman 50 filter paper (2.7 μm particle retention) to obtain the filtrate. To assess boron adsorption efficiency at various pH levels and compare these results with CGC particles, identical experiments were conducted following the same procedures outlined above.

#### 2.4.2. Boron adsorption isotherms

For the adsorption isotherms, 0.8 g of CGCNF beads were immersed in 20 mL of boric acid solution, with initial boron concentrations ranging from 10 to 400 mg L^−1^, within polypropylene bottles. The initial pH solutions were at 5.45. These mixtures were subjected to shaking in a water bath at 25 °C for 24 h to attain equilibrium, followed by filtration through Whatman 50 filter paper to separate CGCNF beads from the filtrate. To benchmark the efficiency of CGCNF beads, the boron adsorption capacities of CGC particles and the commercial resin Amberlite IRA-743 were also investigated under the same conditions. The experimental data were then simulated using the Langmuir, Freundlich, and Temkin models.

#### 2.4.3. Boron adsorption kinetics

In this experiment, 0.8 g of CGCNF beads were suspended in 20 mL of boric acid solution with an initial boron concentration of 400 mg L^−1^. The samples were agitated in a water bath at 25 °C for durations ranging from 15 min to 24 h. All experiments were conducted at a boric acid pH of 5.56. The same experiment was conducted for CGC particles. Filtrate samples were collected using the filtration method, and the experimental data for boron adsorption were simulated using the pseudo-first order, pseudo-second order, and intra-particle diffusion models.

#### 2.4.4. Effect of foreign ions on boron adsorption

To assess boron uptake capacity in the presence of competitive ions, additional adsorption experiments were conducted in boron solutions containing NaCl, KCl, CaCl_2_, or MgCl_2_. Specifically, 0.8 g of CGCNF beads were immersed in 20 mL of boric acid solution with an initial boron concentration of 400 mg L^−1^ in the presence of NaCl, KCl, CaCl_2_, or MgCl_2_. The ion strengths were set at 1.0, 2.0, 3.0, and 4.0 mol L^−1^ for Na^+^, K^+^, and Ca^2+^, and 0.5, 1.0, 1.5, and 2.0 mol L^−1^ for Mg^2+^, respectively. All samples were shaken at 25 °C for 24 h to achieve adsorption equilibrium. At the conclusion of the experiment, filtrate was collected by filtration for the measurement of boron concentrations.

#### 2.4.5. Adsorption efficiency of various metals and metalloids on CGCNF beads

To assess the adsorption efficiency of various metals and metalloids on CGCNF beads, 0.8 g of the beads were introduced into 20 mL solutions containing separately Se(vi), As(iii), As(v), Cr(iii), or Cr(vi). The initial concentrations of 5 samples were set at 400 mg L^−1^, and the experiments were conducted at 25 °C for 24 h. The experiment for CGC particles was performed following the same procedures. After filtration, the concentrations in the samples were determined using an inductively coupled plasma-atomic emission spectrometer (ICP-AES). All the experiments of boron adsorption were carried out in duplicate to ensure accurate results.

### 2.5. Reusability studies

For the reusability studies, CGCNF beads obtained from the filtration step of boron adsorption were washed multiple times with Milli-Q water. Subsequently, the beads were suspended in 50 mL of a 0.1 M HCl solution in a polypropylene bottle and shaken at 25 °C for 24 h. Afterwards, the CGCNF beads underwent several washes with Milli-Q water. To reactivate the adsorbent, the beads were introduced into 50 mL of a 0.1 M NaOH solution, followed by shaking at 25 °C for 12 h. Finally, the beads were dried in a freeze dryer for 12 h for the next adsorption–desorption cycle. The reusability study of CGC particles was carried out the same procedure with 5 cycles.

Blank samples were also conducted for each batch test. The boron concentration in the samples was measured using a UV/VIS/NIR spectrophotometer through the Azomethine-H method. The determination of boron concentration was carried out at a wavelength of 415 nm. The removal efficiency of boron (*H*) and boron adsorption capacity (*q*_e_) can be calculated as follows:1
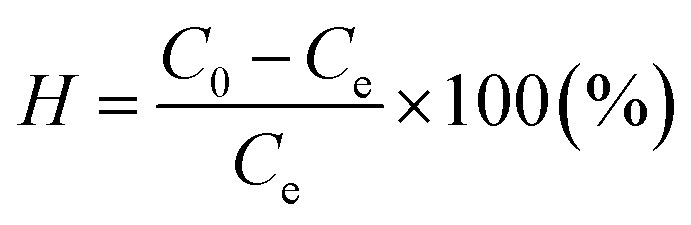
2
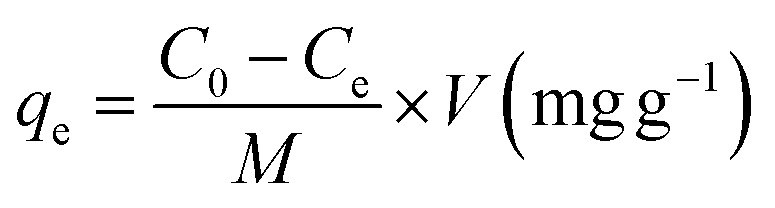
where *C*_0_ and *C*_e_ (mg L^−1^) represent the initial and equilibrium concentrations of boron in the solution, respectively, *M* (g) is the weight of dry adsorbent and *V* (L) is the volume of solution.

## 3 Results and discussion

### 3.1. ^1^H NMR and Cosy NMR analysis

**Fig. 2:**
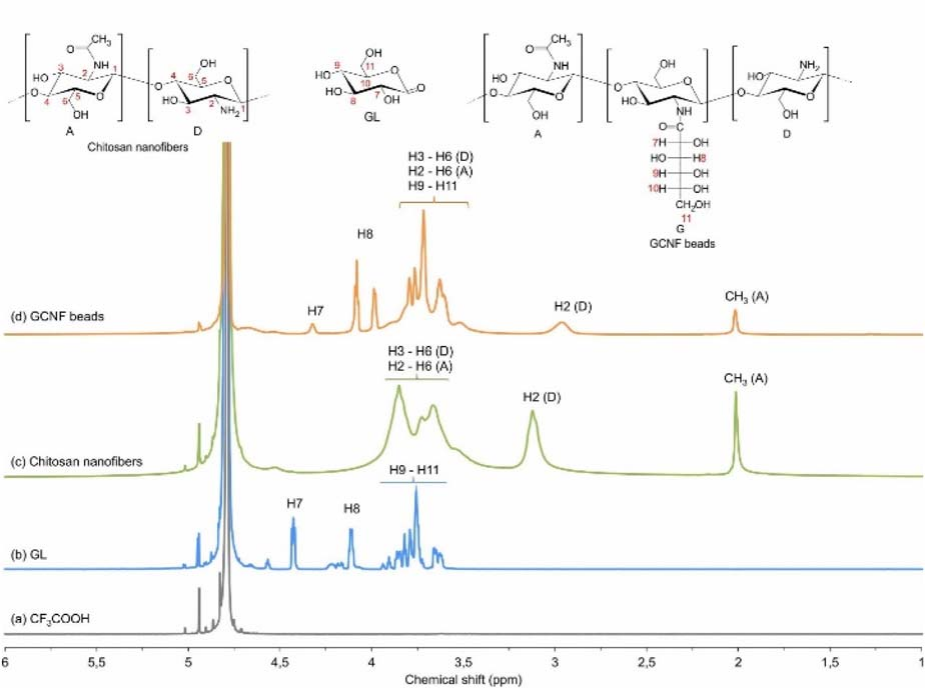
^1^H NMR spectra of different samples (a) CF_3_COOH/D_2_O [2%, *v*/*v*], (b) GL in D_2_O, (c) chitosan nanofibers in CF_3_COOH/D_2_O [2%, *v*/*v*] and (d) GCNF beads in CF_3_COOH/D_2_O [2%, *v*/*v*].

Fig. 2 presents the ^1^H NMR spectra of the solvent CF_3_COOH in D_2_O, GL in D_2_O, chitosan nanofibers, and GCNF beads in CF_3_COOH/D_2_O. The spectra clearly show the overlap of the signal of H1 in acetylated units and deacetylated units with the solvent CF_3_COOH in D_2_O. This finding is similar to the result of other studies.^32^ The internal standard applied for assigning the chemical shifts of the protons was D_2_O/CF_3_COOH, which developed as a peak at 4.79 ppm. Proton assignments for chitosan nanofibers, as reported in our previous work, include *δ*_3.10_ correlated to H2 (deacetylated units), *δ*_2.01_ related to three methyl H atoms (acetylated units), and *δ*_3.68–3.84_ assigned to ring protons from H3 to H6 (deacetylated units) and ring protons from H2 to H6 (acetylated units). Proton assignments for GL were reported as follows: *δ*_4.41_ was attributed to H7, and *δ*_4.10_ corresponded to H8.^30^ Chitosan nanofibers were chemically functionalized with GL through the reaction of amine groups and gluconated groups. The chemical grafting of gluconated units onto the chitosan nanofibers skeleton was confirmed by the ^1^H NMR spectrum, revealing various small peaks (Fig. 2d).

**Fig. 3:**
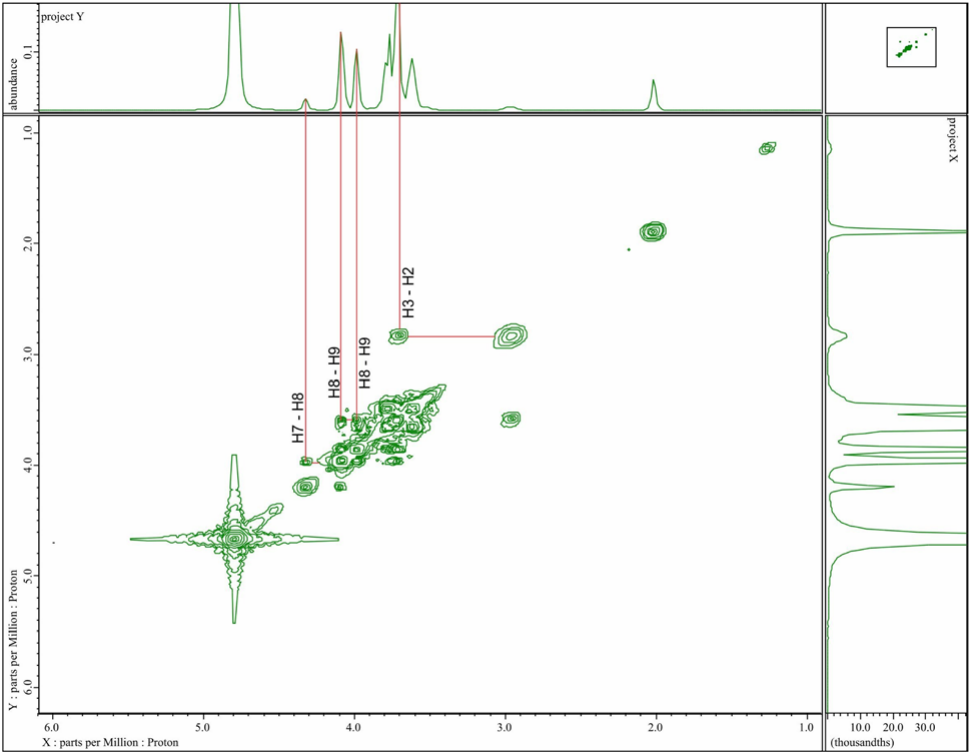
Cosy NMR spectrum of GCNF beads.

The structure of GCNF beads was further elucidated by detailed 2D COSY, specifying the positions of diverse protons on the chitosan nanofibers backbone and protons on gluconated units (Fig. 3). Coupling reactions between H2 and H3, and between H7 and H8, were observed. The new signal of GCNF beads, *δ*_4.32_, was identified as H7. Notably, the new signals at *δ*_4.08_ and *δ*_3.98_ were attributed to H8, both coupled to H9, indicating that H8 was split by H9. In addition, the results of GC particles using chitosan flakes from ^1^H NMR and 2D COSY analysis were similar to GCNF beads using chitosan nanofibers.^30^ The appearance of protons H7 and H8 provides further evidence that gluconated groups were successfully grafted onto chitosan flakes and chitosan nanofibers. However, these methods may not be able to determine the degree of gluconated groups on GC particles and GCNF beads. Therefore, colloidal titration (presented in the next section) was carried out to ascertain the degree of gluconated units and the level of grafted gluconated units on both GC particles and GCNF beads.

### 3.2. Colloidal titration

The presentation of the degree of acetylated units (DA%), DD%, and the degree of gluconated units (DG%) in the structure of chitosan nanofibers and GCNF beads is depicted in Fig. S3.†Table 1 presents the characteristics of chitosan and modified chitosan. In a previous study, the DD% of chitosan flakes and chitosan nanofibers was determined to be 84.65% and 88.11%, respectively.^30^ Based on these values, the calculated DG% for GC particles and GCNF beads was found to be 13.88% and 43.60%, respectively. Details of the calculation are shown in ESI (eqn (S1)–(S4)).† Additionally, the level of grafted gluconted units (LGG%) for GC particles and GCNF beads was 16.4% and 49.5%, respectively. Notably, the level of gluconated units introduced in chitosan nanofibers was observed to be three times higher than in chitosan flakes. This enhancement in the level of gluconated units in GCNF beads is attributed to the high porosity, strong interconnection, small inner fibrous pore size, and adjustable surface functionality (free amine groups) within the structural network of chitosan nanofibers.

**Table 1:** Characteristics of chitosan and modified chitosan^a^

Sample	DD (%)	DG (%)	LGG (%)	Ref.
Chitosan flakes	84.65 ± 0.63	n.a	n.a	30
Chitosan nanofibers	88.11 ± 0.45	n.a	n.a	30
GC particles	70.77 ± 0.33	13.88 ± 0.71	16.4 ± 0.85	This work
GCNF beads	44.51 ± 0.27	43.60 ± 0.69	49.5 ± 0.82	This work

a n.a: not applicable.

### 3.3. The stability of GC particles, CGC particles, GCNF beads and CGCNF beads

The solubility test results for all adsorbents are presented in Table S1.† GC particles and GCNF beads demonstrate insolubility in neutral and alkaline environments, while they readily dissolve in acidic solutions. This property limits the reusability of GC particles and GCNF beads, as the desorption process requires the use of an acid solution to release boron. Following crosslinking with EGDE, CGC particles and CGCNF beads were subjected to suspension in 0.1 M HCl and 6 M HCl solutions for one month. Notably, these adsorbents did not dissolve in different HCl solutions (Fig. S4†). It is suggested that the crosslinking step enhances the chemical durability and mechanical stability of adsorbents in acidic solutions.^33^ Consequently, CGC particles and CGCNF beads can be effectively applied for desorption in acidic media.

### 3.4. SEM analysis and BET surface area

**Fig. 4:**
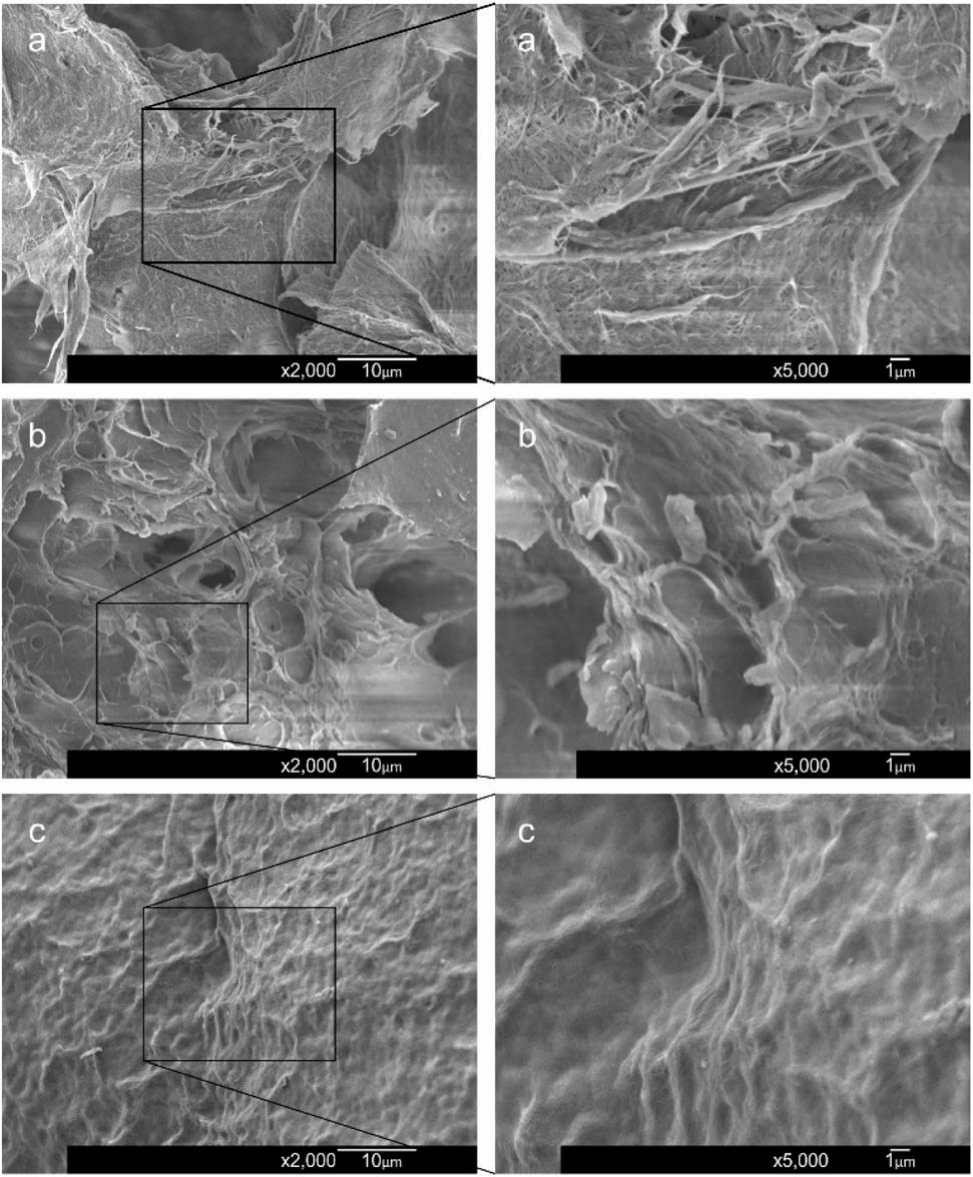
SEM images of chitosan nanofibers (a), CGC particles (b), and CGCNF beads (c) after freeze-drying at different magnifications.

The SEM images in Fig. 4 depict chitosan nanofibers, CGC particles, and CGCNF beads after freeze-drying at various magnifications. The surface morphology of chitosan nanofibers reveals a dense network of fibers (Fig. 4a). In the meanwhile, the CGC sample displays a less porous morphology (Fig. 4b). Following physical and chemical modification, CGCNF beads display a porous and rough morphology (Fig. 4c), contributing to a substantial specific surface area and an increased number of active binding sites. This unique structural characteristic enhances the boron adsorption capacity of CGCNF beads.

In the meanwhile, the N_2_ adsorption–desorption isotherms of both the CGC particles and CGCNF samples exhibit type III behavior, indicating nonporous adsorbents with a H4 type associated with narrow slit pores (Fig. S5†).

The BET surface area of the CGC particles was measured at 3.4 ± 0.3 m^2^ g^−1^ with a total volume pore of 4.1 × 10^−3^ ± 4.0 × 10^−4^ cm^3^ g^−1^, while that of the CGCNF beads was significantly higher at 20.9 ± 2.1 m^2^ g^−1^ with total volume pore of 1.6 × 10^−2^ ± 1.4 × 10^−3^ cm^3^ g^−1^. Therefore, the chemical interactions between boron and CGCNF involve dominantly chemical bonding rather than physical adsorption, which will be discussed in the next section. The pore size distribution of CGCNF sample is also presented in Fig. S6,† confirming its nonporous materials. The irregularity of the bead surface observed in SEM images (Fig. 4) contributes to the larger specific surface area of the CGCNF beads. However, despite this irregularity, the porosity may be reduced due to the more compact chemical structure caused by cross-linking. Additionally, the blockage of internal chitosan pores by gluconated molecules could be another probable reason for the observed low in porosity.

### 3.5. TGA

Fig. S7† presents the TGA results of the prepared samples. The initial sharp reduction in the mass of CGCNF beads and CGC particles observed within the first 150 °C of the TGA experiments is attributed to the evaporation of moisture and humidity. Following this initial loss, the CGCNF beads exhibited stability up to 250 °C, whereas the CGC particles showed a sharp decline. This result aligns well with the N_2_ adsorption–desorption isotherm data, indicating that CGC particles have larger surface areas, which leads to greater moisture absorption and its continuous evaporation as the temperature increases. From 250 °C onwards, both samples showed a continuous weight decrease with increasing temperature. The CGCNF beads were completely decomposed by 820 °C, whereas the CGC particles retained about 20% of their weight at this temperature. The TGA results indicate that CGCNF beads and CGC particles behave differently under thermal conditions due to differences in their physical properties. The slightly larger surface area of CGC particles leads to higher moisture absorption, causing a more gradual mass loss compared to CGCNF beads. The CGCNF beads' stability up to 250 °C suggests better thermal resistance in this range, while the complete decomposition by 820 °C reflects their overall lower thermal stability compared to CGC particles, which retain residual mass due to potentially more stable components or structures within the material. The stability of CGCNF beads up to 250 °C and their mass loss starting at a higher temperature is not an issue for the boron removal experiment; in fact, it is a desirable property. This indicates that the CGCNF beads are suitable for boron removal from wastewater, as the process is typically performed at room temperature, well below 250 °C, both in laboratory settings and practical applications. In contrast, CGC particles display continuous weight loss within this temperature range, making CGCNF beads a more promising adsorbent for boron removal for future development and applications.

### 3.6. FTIR

**Fig. 5:**
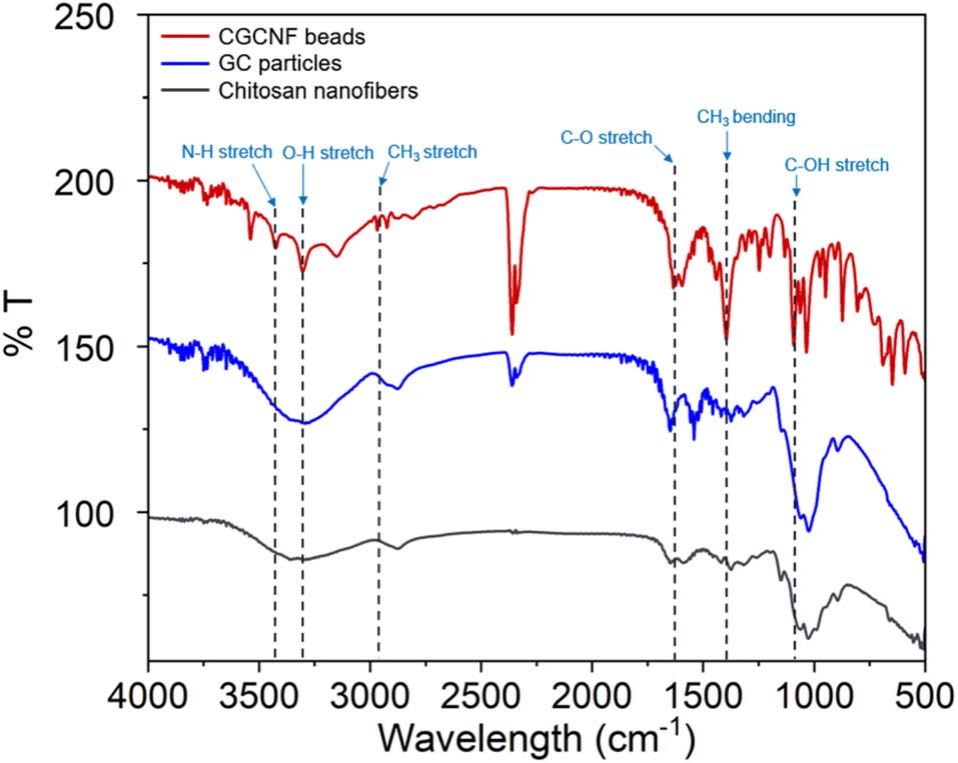
FTIR of chitosan nanofibers, GC particles, and CGCNF beads.

The FTIR spectra of chitosan nanofibers, GC particles, and CGCNF beads are shown in Fig. 5. Key peaks for chitosan nanofibers include 3439 cm^−1^ (N–H and O–H stretching), 2925 cm^−1^ (CH_3_ symmetric stretch), 1666 cm^−1^ (C–O stretch), and 1073 cm^−1^ (C–OH stretch). In the case of chitosan nanofibers and GC particles, the N–H stretching overlaps with the broad O–H peak, making it difficult to distinguish them clearly. However, for CGCNF beads, the N–H and O–H peaks are well-separated and distinct. This distinction occurs due to the crosslinking process, which alters the molecular structure and strengthens the separation between these two functional groups, resulting in sharper, more defined peaks. In CGCNF beads sample, shifts are observed: the N–H and O–H stretch moves from 3439 to 3417 cm^−1^, CH_3_ stretch from 2925 to 2937 cm^−1^, C–O stretch from 1666 to 1645 cm^−1^, C–N stretch from 1438 to 1406 cm^−1^, and C–OH stretch from 1073 to 1037 cm^−1^. These results align with prior studies,^34,35^ suggesting that the presence of –OH functional groups could promote boron adsorption. Such –OH groups could form strong stable complexes with boron species.

### 3.7. Effect of initial pH values on boron adsorption

**Fig. 6:**
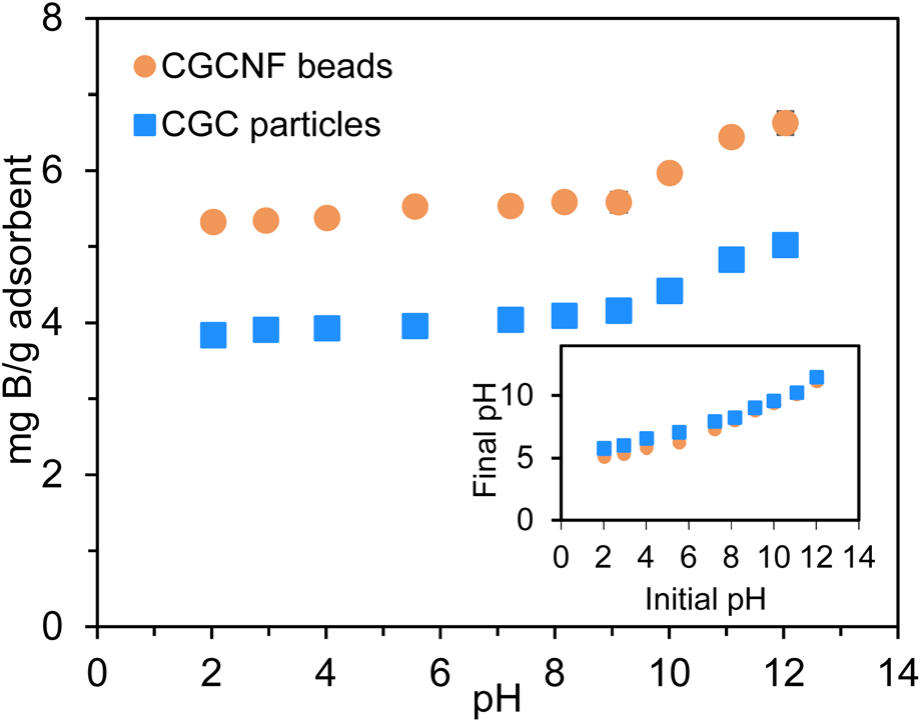
Effect of initial pH solution on boron adsorption by using CGCNF beads and CGC particles. Inset graph shows the final pH after boron adsorption using CGCNF beads (initial concentration of boron: 400 mg L^−1^, mass of adsorbent: 0.8 g, initial pH solution: 2.03–12.03, solution volume: 20 mL, contact time: 24 h and temperature: 25 °C).

pH is a critical parameter that significantly influences the adsorption efficiency of adsorbents. Fig. 6 illustrates the impact of pH values on the boron adsorption capacity of CGCNF beads and CGC particles, and the inset figure exhibits the final pH after boron adsorption. The pH investigation for boron adsorption covered a range from 2.03 to 12.03. It is evident that the boron adsorption capacity of both CGCNF beads and CGC particles gradually increased as the initial pH of the solution rose from 2.03 to 10.01, and it significantly increased between initial pH values of 10.01 and 12.03. At the optimal pH of 12.03, the highest boron adsorption capacity reached 6.63 and 5.02 mg g^−1^, with maximum boron removal efficiencies of 65.61% and 49.66%, using CGCNF beads and CGC particles, respectively (Fig. S8†). Notably, CGCNF beads and CGC particles exhibited superior performance for boron removal at higher pH values. This phenomenon can be attributed to the surface charge of the adsorbents. As the initial pH of the solution increased, the higher adsorption efficiency at alkaline conditions is attributed to the presence of excess hydroxyl ions in gluconated groups. These hydroxyl ions can react with boron, forming strong borate complexes. Furthermore, boric acid B(OH)_3_ exists mainly in aqueous solution at pH < 6 while polyborate species such as [B_3_O_3_(OH)_4_]^−^, [B_4_O_5_(OH)_4_]^2−^ and [B_5_O_6_(OH)_4_]^−^ are predominantly formed in the range of pH value from 6 to 10. When pH is higher than 10, [B(OH)_4_]^−^ ion exists chiefly in aqueous solution.^36^ All B(OH)_3_, polyborate species and [B(OH)_4_]^−^ ions could generate complexes with *vis*-diols; however, tetrahedral boron coordination structure created by *vis*-diols and [B(OH)_4_]^−^ is much stronger than B(OH)_3_.^37^ The enhancement in boron adsorption efficiency with increasing pH can be attributed to the intensified interaction between *vis*-diols on the adsorbent surface and boron. The rise in pH promotes this interaction. Conversely, as the initial pH decreases, the positively charged surfaces of CGCNF beads and CGC particles increase due to protonation. This leads to the formation of repulsive forces between functional groups on the adsorbent surface and boron, resulting in decreased adsorption efficiency. The findings suggest that the adsorbents can be activated by immersion in an alkaline solution before the adsorption process and can be regenerated through acid treatment. Analyzing the trend depicted in Fig. 6, it becomes apparent that raising the pH beyond 12 has the potential to enhance boron adsorption capacity. However, it is crucial to emphasize the practical considerations relevant to wastewater treatment applications. The use of NaOH for pH adjustment, particularly at higher pH levels, raises safety concerns due to its corrosive nature, posing risks not only to human eyes, skin, and respiratory systems but also to pipeline systems upon contact. Additionally, it may contribute to the creation of secondary toxic contaminants. Striking a balance between achieving effective boron removal and ensuring the safety and practicality of the treatment process is imperative in practical applications.

### 3.8. Boron adsorption isotherm

Isotherm data is significant for predicting the adsorption mechanism and adsorption capacity of a variety of adsorbents.^38^ In this study, the experimental data were simulated *via* Langmuir, Freundlich, and Temkin adsorption isotherm models. The non-linear form of Langmuir isotherm equation is represented as follows:3
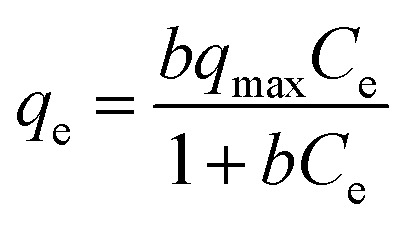


The linear form of Langmuir isotherm equation is described as follows:4
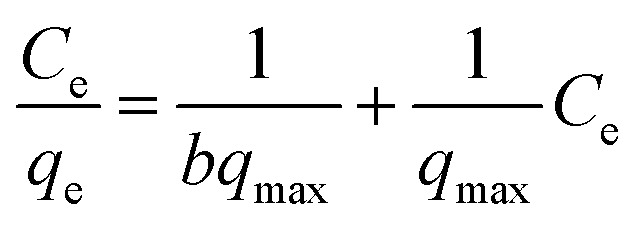
where *q*_max_ is the highest adsorption capacity (saturation value) (mg g^−1^ adsorbent), *q*_e_ is the amount of adsorbed boron at equilibrium in the solution (mg g^−1^ adsorbent), *C*_e_ is the boron concentration at equilibrium in solution (mg L^−1^), and *b* is the Langmuir constant involved in energy of adsorption (L mg^−1^ adsorbent).

The non-linear form of Freundlich isotherm equation is as follows:

5 *q*_e_ = *K*_F_*C*_e_^1/*n*^

The linear form of Freundlich isotherm equation is given as follows:6
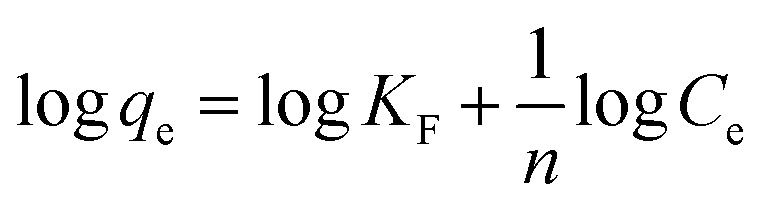
where *K*_F_ is the Freundlich constants and *n* is the equilibrium coefficient, respectively.

The non-linear form of Temkin isotherm equation is as follows:

7 *q*_e_ = *B* ln(*A*_T_*C*_e_)

The linear form of Temkin isotherm equation can be expressed as follows:

8 *q*_e_ = *B* ln(*A*_T_) + *B* ln(*C*_e_)

and

9 *B* = *RT*/*b*_T_

where *b*_T_ is the constant of Temkin isotherm (kJ g mol^−2^), *R* is the ideal gas constant (8.3145 J mol^−1^ K^−1^), *T* is thermodynamic temperature (K), and *A*_T_ represents the constant of equilibrium binding corresponding to the maximum binding energy (L g^−1^).

**Fig. 7:**
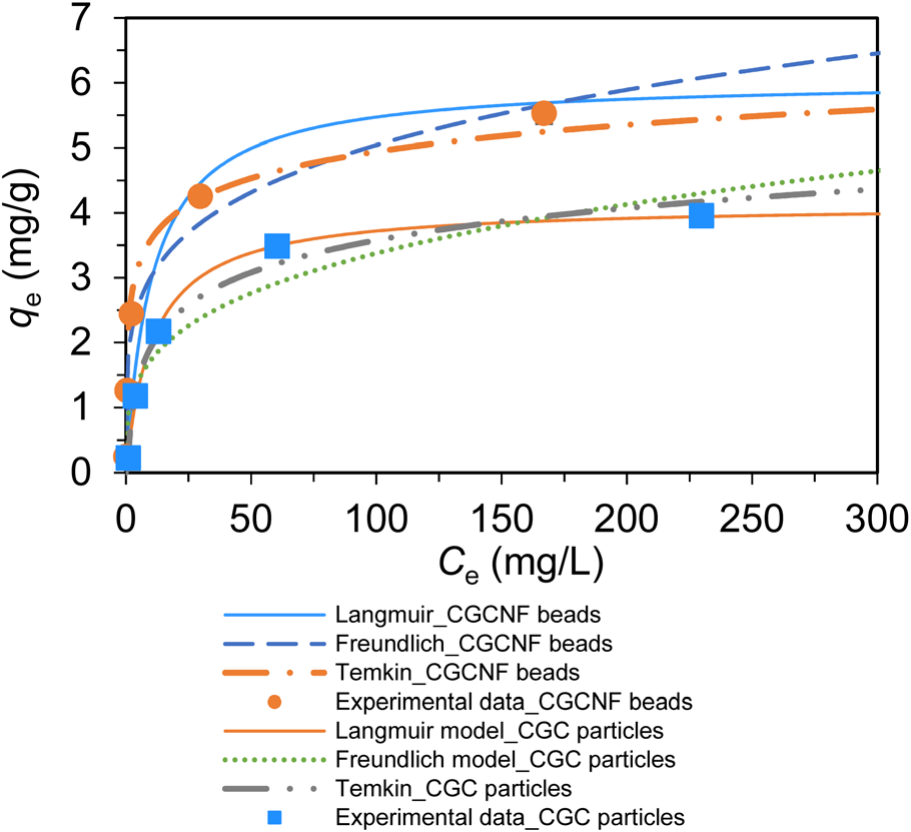
Fitting of boron adsorption isotherm models onto CGCNF beads and CGC particles (initial concentration of boron: 10–400 mg L^−1^, mass of adsorbent: 0.8 g, initial pH solution: 5.45, solution volume: 20 mL, contact time: 24 h and temperature: 25 °C).

The calculated parameters and fitted results of these three isotherm models are given in Table S2† and Fig. 7, respectively. The plots of *C*_e_/*q*_e_*versus C*_e_, log(*q*_e_) *versus* log(*C*_e_), and *q*_e_*versus* ln(*C*_e_) for the adsorption of boron onto CGCNF beads and CGC particles formed according to the linear forms of Langmuir, Freundlich, and Temkin isotherms are shown in Fig. S9 and S10,† respectively.

As can be seen from Table S2,† the *R*^2^ value of Langmuir adsorption isotherm of CGCNF beads is 0.9976, which is higher than 0.9441 of Freundlich, and 0.9687 of Temkin adsorption isotherm models. The findings suggest that Langmuir adsorption isotherm model is well-suited to describe the boron adsorption onto CGCNF beads. These results imply that the adsorption process follows a monolayer and homogeneous mechanism. Additionally, the use of the dimensionless separation factor *R*_L_ in Langmuir isotherm aids in predicting the favorability of the boron adsorption process by CGCNF beads.^39^ The equation for the determination of *R*_L_ is presented as follows:10
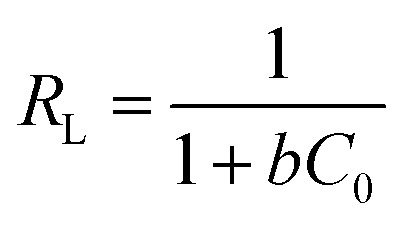
where *C*_0_ is the initial boron concentration in the solution (mg L^−1^). *R*_L_ refers to boron adsorption types including favorable (0 < *R*_L_ < 1), unfavorable (*R*_L_ > 1), linear (*R*_L_ = 1) and irreversible (*R*_L_ = 0). As shown in Table S2,† the values of *R*_L_ in the range of 0.027–0.513 less than 1 suggest that the adsorption process of boron onto CGCNF beads is favorable.

A list of the maximum adsorption capacities for boron onto CGCNF beads and other adsorbents reported in previous work is shown in Table 2. According to Langmuir adsorption isotherm, the *q*_max_ of CGCNF beads (*q*_max_ = 6.05 mg g^−1^) for boron removal from this work is higher than CGC particles (*q*_max_ = 4.13 mg g^−1^) and other adsorbents. In order to assess the practicability of CGCNF beads in this work, the boron adsorption process using commercial resin Amberlite IRA-743 was performed under the same conditions. Interestingly, the *q*_max_ of CGCNF beads is higher than that of this commercial resin (5.73 mg g^−1^). Thus, CGCNF beads adsorbent is a suitable and promising material for the removal of boron from aqueous solution since it possesses higher adsorption capacity than other adsorbents.

**Table 2:** Comparison of maximum adsorption capacities of boron onto various adsorbents

Adsorbent	*q*_max_ (mg g^−1^)	Experimental conditions	Ref.
Granular activated carbon	0.85	*C*_0_ = 5–60 mg L^−1^, pH = 7, 25 °C, 4 h, dose = 0.5 g/50 mL	11
Granular activated carbon modified with mannitol	1.50	*C*_0_ = 5–60 mg L^−1^, pH = 8.5, 25 °C, 4 h, dose = 0.5 g/50 mL	
Granular activated carbon modified with xylitol	1.45	*C*_0_ = 5–60 mg L^−1^, pH = 8.5, 25 °C, 4 h, dose = 0.5 g/50 mL	
Granular activated carbon modified with sodium gluconate	1.04	*C*_0_ = 5–60 mg L^−1^, pH = 8.5, 25 °C, 4 h, dose = 0.5 g/50 mL	
Calcined alunite	3.39	*C*_0_ = 5–250 mg L^−1^, pH = 10, 25 °C, 48 h, dose = 1 g/25 mL, 140 rpm	40
Rice hulk	5.00	*C*_0_ = 50–300 mg L^−1^, pH = 5–10, 25 °C, 8 h, dose = 1 g/100 mL, 90 rpm	41
Chelating resins with pyrocatechol functional group	4.54	*C*_0_ = 22–108 mg L^−1^, 25 °C, 24 h, dose = 1 g/100 mL	42
T_i_O_2_-chitosan beads	4.12	*C*_0_ = 0.5–20 mg L^−1^, pH = 4, 25 °C, 5 min, dose = 1 g/20 mL	43
Cr_2_O_3_-chitosan beads	3.66	*C*_0_ = 0.5–20 mg L^−1^, pH = 4, 25 °C, 5 min, dose = 1 g/20 mL	
Fe_3_O_4_-chitosan beads	4.38	*C*_0_ = 0.5–20 mg L^−1^, pH = 4, 25 °C, 5 min, dose = 1 g/20 mL	
Fe(OH)_3_-chitosan beads	11.10	*C*_0_ = 0.5–20 mg L^−1^, pH = 4, 25 °C, 5 min, dose = 1 g/20 mL	
Chitosan particles	—	*C*_0_ = 10–400 mg L^−1^, pH = 5.45, 25 °C, 24 h, dose = 0.8 g/20 mL	This work
Chitosan nanofibers beads	—	*C*_0_ = 10–400 mg L^−1^, pH = 5.45, 25 °C, 24 h, dose = 0.8 g/20 mL	
Amberlite IRA-743	5.73	*C*_0_ = 10–400 mg L^−1^, pH = 5.45, 25 °C, 24 h, dose = 0.8 g/20 mL	
CGC particles	4.13	*C*_0_ = 10–400 mg L^−1^, pH = 5.45, 25 °C, 24 h, dose = 0.8 g/20 mL	
CGCNF beads	6.05	*C*_0_ = 10–400 mg L^−1^, pH = 5.45, 25 °C, 24 h, dose = 0.8 g/20 mL	

### 3.9. Boron adsorption kinetics

Prediction of the adsorption rate for boron removal plays an important role in designing treatment equipment in the practice.^44^ The dynamic adsorption of boron onto CGCNF beads was conducted by pseudo-first order, pseudo-second order, and intra-particle diffusion models.

The pseudo-first order kinetic model is written as the following equation:11
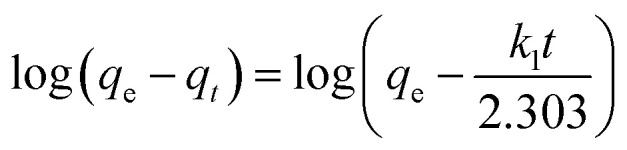


The non-linear form of pseudo-first order model is expressed as follows:

12 *q*_*t*_ = *q*_e_(1 − exp(−*k*_1_*t*))

The pseudo-second order kinetic model is given as follows:13
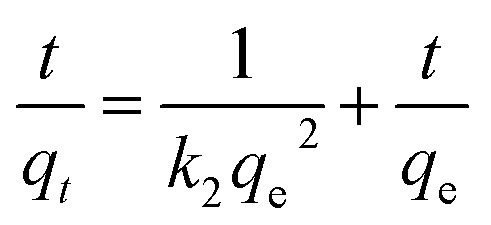


The non-linear form of pseudo-second order model is presented as follows:14
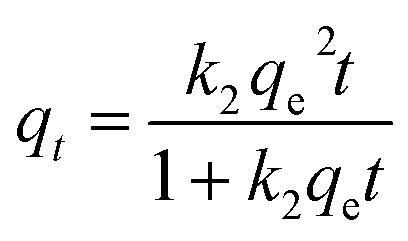


The intra-particle diffusion model can be expressed by the following equation:

15 *q*_*t*_ = *K*_diff_*t*^1/2^ + *C*

where *q*_*t*_ is the adsorbed amounts of boron onto adsorbent (mg g^−1^ adsorbent) at time *t*, *q*_e_ is the adsorbed amounts of boron onto adsorbent at equilibrium (mg g^−1^ adsorbent), *k*_1_ (min^−1^) is the rate constant of pseudo-first order model, *k*_2_ (g mg^−1^ min^−1^) is the rate constant of the pseudo-second order model, *K*_diff_ is the diffusion rate constant (mg g^−1^ min^−1/2^), and *C* is intra-particle diffusion constant intercept of the line (mg g^−1^).

**Fig. 8:**
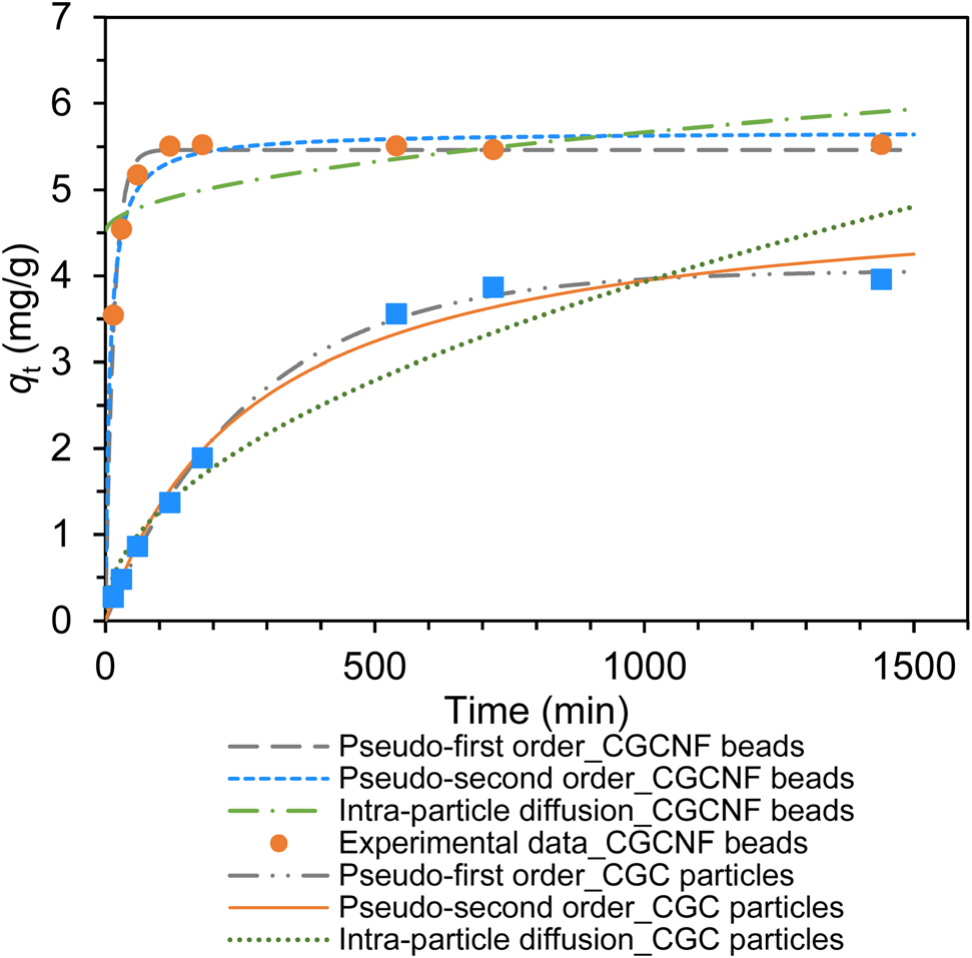
Fitting of boron adsorption kinetic models onto CGCNF beads and CGC particles (initial concentration of boron: 400 mg L^−1^, mass of adsorbent: 0.8 g, initial pH solution: 5.56, solution volume: 20 mL, contact time: 0–24 h and temperature: 25 °C).

The plots of the pseudo-first-order, pseudo-second-order, and intra-particle diffusion kinetics of boron adsorption and kinetics parameters are shown in Fig. S11, S12 and Table S3.† It is found that the correlation coefficient *R*^2^ of CGCNF beads obtained from the pseudo-second order kinetic model (*R*^2^ = 1.0000) was higher than that obtained from the pseudo-first order kinetic model (*R*^2^ = 0.9958) and intra-particle diffusion (*R*^2^ = 0.3860). The findings suggest that the adsorption of boron ions onto CGCNF beads is likely controlled by chemical adsorption through the exchange of boron ions and *vis*-diol functional groups. As depicted in Fig. 8, the kinetic curve for boron onto CGCNF beads indicates rapid initial adsorption, reaching equilibrium after 120 minutes.

In contrast, CGC particles obtained the adsorption equilibrium time after 720 minutes. These results highlight the crucial role of the structural properties of modified chitosan nanofibers, including surface area and porosity, in enhancing the adsorption rate compared to modified chitosan flakes.

### 3.10. Effect of foreign ions on boron adsorption

**Fig. 9:**
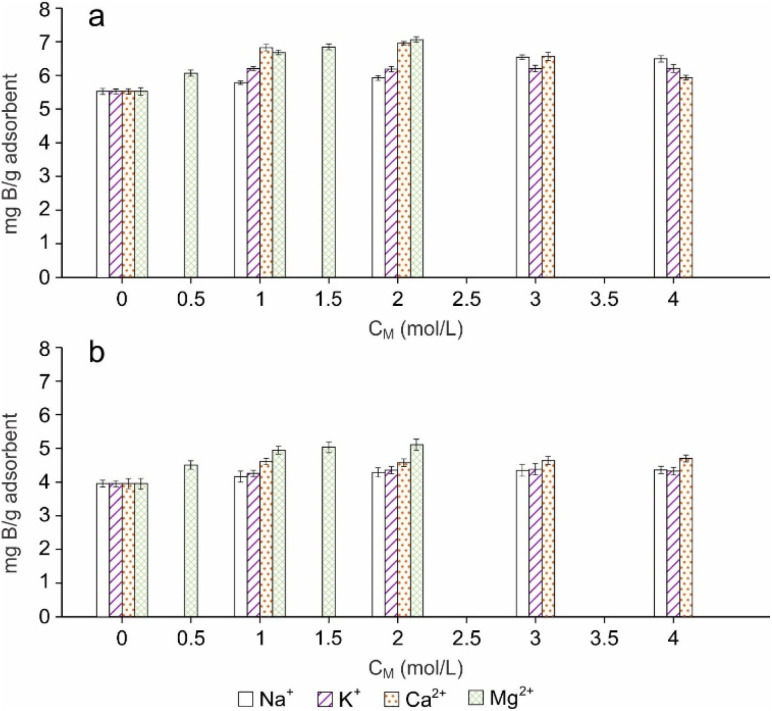
Effect of ions Na^+^, K^+^, Ca^2+^ and Mg^2+^ on boron adsorption capacity by using (a) CGCNF beads and (b) CGC particles.

In order to examine the interference of competitive ions on boron adsorption, experiments were conducted in the presence of Na^+^, K^+^, Ca^2+^ and Mg^2+^. The effect of foreign ions on boron adsorption is given in Fig. 9 and S13.† Boron adsorption capacity of CGCNF beads slightly increased, and the highest boron adsorption capacity was achieved at 6.49 and 6.21 mg g^−1^ in the presence of Na^+^ and K^+^, respectively, comparing boron adsorption capacity at 5.53 mg g^−1^ without foreign ions. The maximum boron removal efficiency was 62.2 and 59.9% with the addition of Na^+^ and K^+^. Furthermore, the rapid enhancement in boron adsorption capacity was observed in the presence of Ca^2+^ and Mg^2+^ ions. The peak boron adsorption capacity reached 6.96 and 7.07 mg g^−1^, with maximum removal efficiencies of 66.1% and 70.8%. This improved performance is likely attributed to the salting-out effect, as documented in previous studies.^45,46^ The significant increase in boron adsorption in the presence of Ca^2+^ and Mg^2+^ ions is attributed to two hydroxyl groups bonding to Ca^2+^ and Mg^2+^ ions playing as *vis*-diol groups, which can form stable complexes with boron in aqueous solution. In addition, similar observations have been reported in previous works.^37^

**Fig. 10:**
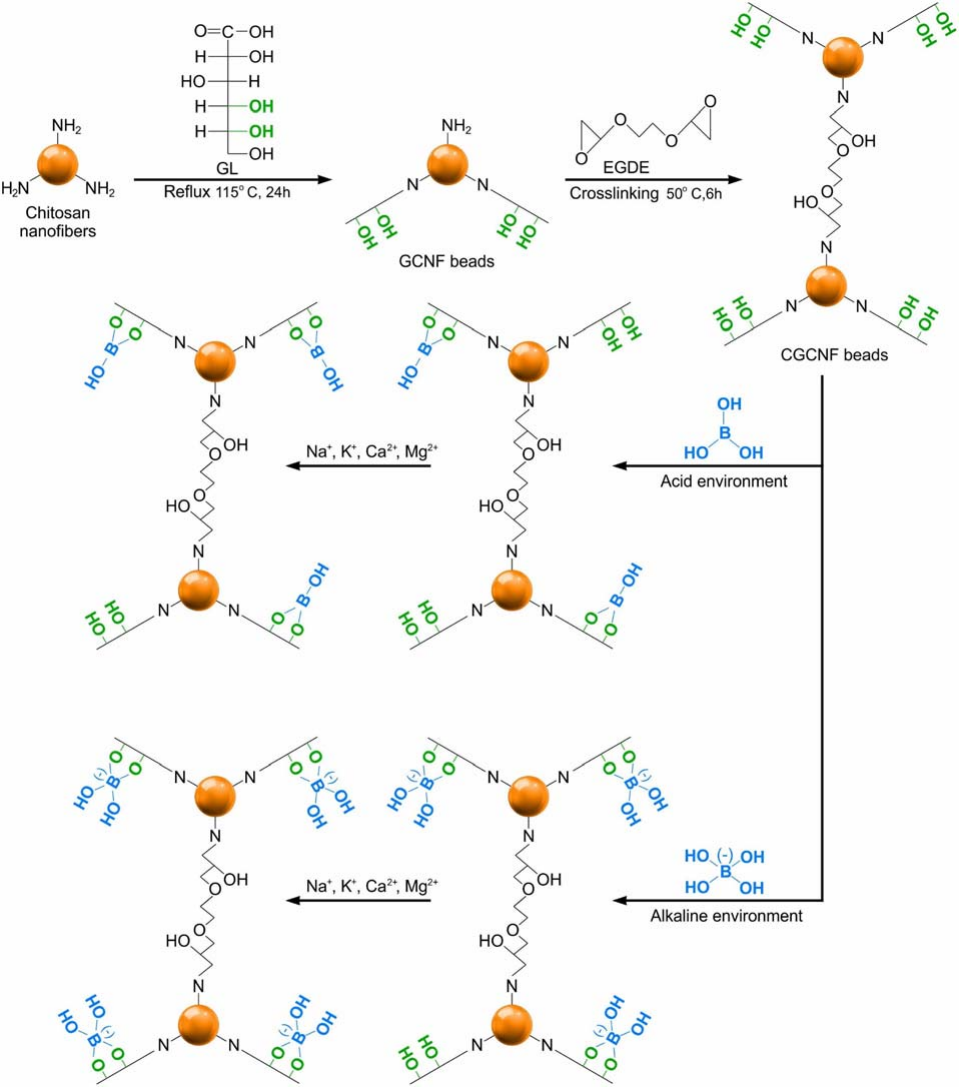
Schematic diagram of the synthesis of CGCNF beads and feasible boron adsorption.

Based on the experimental results discussed above, we propose a potential boron adsorption mechanism as presented in Fig. 10. Under acidic conditions, where most boron exists as H_3_BO_3_, protonation of amine and hydroxyl groups weakens chelation, hampering adsorption. Boric acid's molecular form changes with aqueous pH, impacting adsorption. As mentioned earlier, boric acid B(OH)_3_ exists mainly in aqueous solution at pH < 6 while polyborate species such as [B_3_O_3_(OH)_4_]^−^, [B_4_O_5_(OH)_4_]^2−^ and [B_5_O_6_(OH)_4_]^−^ are predominantly formed in the range of pH value from 6 to 10, enhancing polyhydroxyl group coordination. When pH is higher than 10, [B(OH)_4_]^−^ ion exists chiefly in aqueous solution and increased solution alkalinity weakens adsorption due to electrostatic repulsion between negatively charged CGCNF bead's surface and [B(OH)_4_]^−^. The presence of more hydroxyl ions competes with boron for adsorbent sites, challenging boron complexation. However, boron –OH– competition is less pronounced, as complexed boric acid resists desorption in alkaline eluents. It is noteworthy that wastewater from wet FGD system of coal-fired power contains high concentration of cations (Ca^2+^, Mg^2+^, Na^+^, and K^+^).^47,48^ Commonly employed in FGD, wet scrubbers use limestone (CaCO_3_) or lime (Ca(OH)_2_) as adsorbents to capture and transform SO_2_ gases. Given this, CGCNF beads emerge as a fitting adsorbent for boron removal applicable in FGD wastewater, brine, or seawater, containing diverse salts like NaCl, KCl, CaCl_2_, and MgCl_2_.

### 3.11. Adsorption efficiency of various metals and metalloids on CGCNF beads and CGC particles

The selective capture properties of CGCNF beads and CGC particles were evaluated through batch experiments examining the adsorption efficiency of various metals and metalloids prevalent in the wastewater from FGD system of coal-fired power plants, including Se(vi), As(iii), As(v), Cr(iii), and Cr(vi).^49^ Notably, Fig. S14† reveals limited effectiveness in removing Se(vi), As(v), and Cr(vi) by CGCNF beads and CGC particles in aqueous solution. In contrast, the CGCNF beads showcases significant adsorption capacities, specifically 3.50 mg g^−1^ for As(iii) and 4.87 mg g^−1^ for Cr(iii), and results of CGC particles were 1.55 mg g^−1^ for As(iii) and 3.38 mg g^−1^ for Cr(iii) at an initial concentration of 400 mg L^−1^. These outcomes underscore the selective capture properties, indicating the formation of stable tetrahedral structures through the binding of trivalent cations and [B(OH)_4_]^−^ ions, resulting in robust complexes. This distinctive phenomenon enhances the adsorption of trivalent cations on *vis*-diol grafted-CGCNF beads, thereby exemplifying the ability of CGCNF beads to selectively and effectively remove trivalent metals and metalloids, including B(iii), As(iii), and Cr(iii), from aqueous solutions.

### 3.12. Regeneration and reusability of CGCNF beads and CGC particles

Desorption and reusability are pivotal considerations for practical applications, impacting operational costs. To assess the potential for practical use, the adsorption–desorption–regeneration cycles were performed 20 times for CGCNF beads and 5 times for CGC particles. The recovery of adsorbents involved using a 0.1 M HCl solution, followed by reactivation using a 0.1 M NaOH solution. This robust performance highlights the efficiency and recyclability of the adsorbents, contributing to cost-effective water treatment solutions. The obtained findings are illustrated in Fig. S15.†

The boron adsorption percentage of CGCNF beads diminishes with increasing cycles but remains above 91.6% after 4 cycles, and over 84.8% after eight cycles, gradually decreasing to 65.1% after 20 cycles. The results obtained in our study exhibited comparative performance with previous findings from other researchers (Table S4†), who have reported a similar trend of boron adsorption decreasing sharply after 6–7 cycles. This consistency across studies underscores the importance of our findings and highlights the reproducibility of the observed phenomenon. This work, therefore, contributes to the body of knowledge in this field by providing further evidence and insights into the behavior of boron adsorption over multiple cycles. The boron adsorption capacity drops from 5.53 to 3.60 mg g^−1^. CGCNF beads exhibit exceptional reusability, maintaining high efficiency through multiple cycles. The 2 to 4 mm bead diameter streamlines filtration, eliminating the need for fine-pore filter paper. Overall, these results highlight the efficiency of CGCNF beads as a reliable adsorbent for sustained boron removal.

### 3.13. Practical application: efficient removal of boron from local wastewater using CGCNF beads and CGC particles

**Fig. 11:**
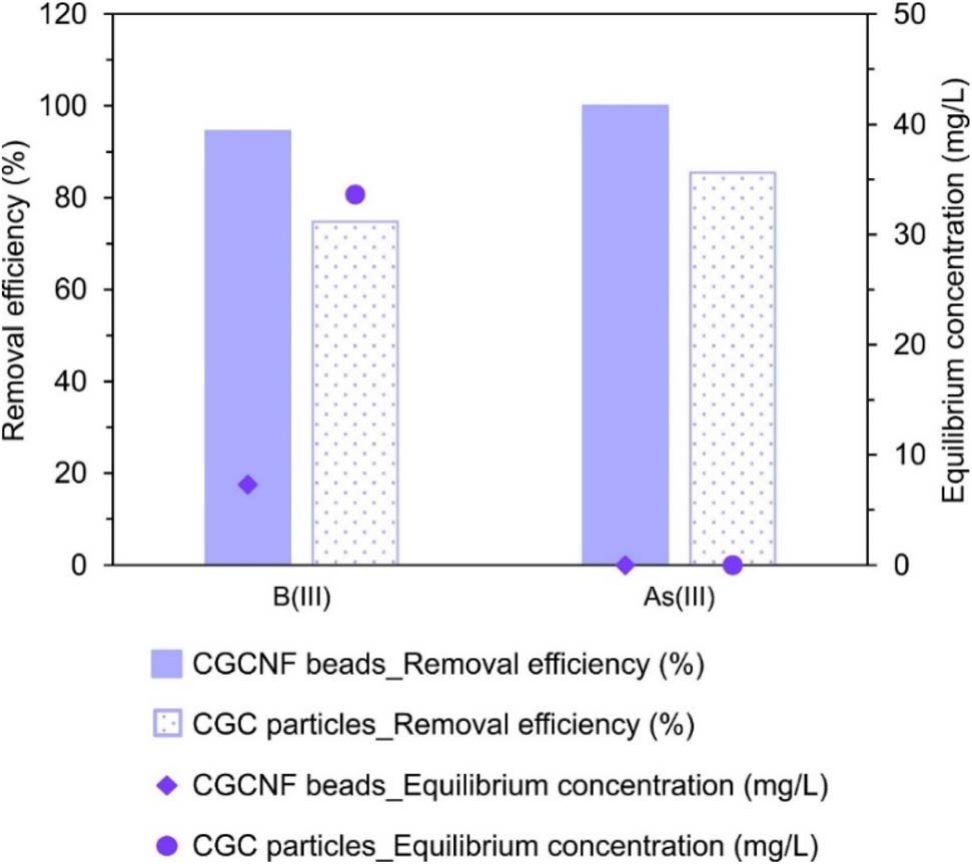
Adsorption efficiency of B(iii) and As(iii) from FGD wastewater of coal-fired power plants by CGCNF beads and CGC particles (equilibrium concentration of CGCNF beads sample: lower limit of detection).

A wastewater sample was collected from FGD system of the local coal-fired power plant (Thanh Hoa province, Viet Nam) and used as influent wastewater to investigate the adsorption efficiency in practice. The samples were filtered through filter paper (0.45 μm pore size) to remove total suspended solids (TSS). In the adsorption experiment, 0.8 g of each adsorbent was added to 20 mL of FGD wastewater. Details of the parameters of FGD wastewater are shown in Table S5.† The mixture was shaken at 25 °C for 24 h. B(iii) and As(iii) concentrations were analyzed by ICP-AES. After the adsorption process using CGCNF beads, the concentration of boron was decreased from 133.29 mg L^−1^ to 7.28 mg L^−1^, and the final concentration of As(iii) was not detected (Fig. 11). The removal efficiency of B(iii) and As(iii) was 94.5% and 100% using CGCNF beads, and 74.8% and 85.5% using CGC particles, respectively. The high concentrations of ions Na^+^, Ca^2+^ and Mg^2+^ in the FGD wastewater may raise the adsorption capacity of B(iii) and As(iii). The results demonstrate that CGCNF beads are an effective adsorbent for the removal of B(iii) and As(iii) from FGD wastewater of coal-fired power plants.

## 4 Conclusions

This work of hydroxyl-functionalized CGCNF beads represents a promising advancement in water treatment, particularly for targeted metal removal. The irregular and porous morphology of these beads enhances the surface area, leading to high boron adsorption capacity supported by Langmuir isotherm and pseudo-second order kinetics. Optimal adsorption occurs at pH 12.03, achieving equilibrium within 120 minutes. Demonstrating robust reusability with 65.1% adsorption retention after 20 cycles underscores their practicality. Both experimental and theoretical findings validated our hypothesis that crosslinking chitosan nanofibers with enhanced –OH groups offer significant advantages, such as direct modification without acid dissolution and improved resistance to acids for prolonged reuse. The –OH groups play a pivotal role in boron removal by forming robust coordination complexes with boron ions, thereby boosting adsorption capacity and selectivity. Beyond boron, CGCNF beads effectively remove As(iii) and Cr(iii) from aqueous solution, positioning them as promising candidates for broader environmental remediation. Future research should focus on optimizing crosslinking strategies and integrating CGCNF beads into practical water treatment systems to maximize their sustainable impact.

## Data availability

Data are available upon request from the authors.

## Author contributions

Ho Hong Quyen: experiment, writing – original draft, data curation, funding acquisition. Hoang M. Nguyen: calidation, writing – review & editing. Vu Chi Mai Tran: data curation, validation. Phuoc-Cuong Le: validation, resources. Masashi Kurashina: experimental supervision, formal analysis. Mikito Yasuzawa: experimental design, data curation. Yuki Hiraga: experiment, data curation, resources.

## Conflicts of interest

There are no conflicts to declare.

## Supplementary Material

RA-015-D5RA00077G-s001

## References

[cit1] de Lucena I. O., de Gois J. S., Cassella R. J. (2024). RSC Adv..

[cit2] Lin J.-Y., Shih Y.-J., Hsieh T.-Y., Huang Y.-H. (2016). RSC Adv..

[cit3] Edition F. (2011). WHO Chron..

[cit4] El-Gohary R. M., El-Shafai N. M., El-Mehasseb I. M., Mostafa Y. S., Alamri S. A., Beltagi A. M. (2024). J. Environ. Manage..

[cit5] Han L., Tian J., Liu C., Lin J., Chew J. W. (2021). Sep. Purif. Technol..

[cit6] Yilmaz A. E., Boncukcuoğlu R., Bayar S., Fil B. A., Kocakerim M. M. (2012). Korean J. Chem. Eng..

[cit7] Chorghe D., Sari M. A., Chellam S. (2017). Water Res..

[cit8] Dong Y., Wang Q., Zhu J., Liang L., Xu D., Mi X., Ren Z., Wang P. (2024). J. Environ. Manage..

[cit9] Yıldırım K., Kasım G. Ç. (2018). Chemosphere.

[cit10] Figueira M., Srivastava V., Reig M., Valderrama C., Lassi U. (2024). J. Environ. Manage..

[cit11] Kluczka J., Pudło W., Krukiewicz K. (2019). Chem. Eng. Res. Des..

[cit12] Polowczyk I., Ulatowska J., Koźlecki T., Bastrzyk A., Sawiński W. (2013). Desalination.

[cit13] Karahan S., Yurdakoç M., Seki Y., Yurdakoç K. (2006). J. Colloid Interface Sci..

[cit14] Yang F., Zhang X., Yao Z. (2022). ACS Appl. Electron. Mater..

[cit15] Sun H., Wang L., Zhang Y., Wang T., Yin X. (2023). Appl. Surf. Sci..

[cit16] Chen T., Lyu J., Wang Q., Bai P., Wu Y., Guo X. (2021). J. Mater. Sci..

[cit17] Jalali M., Rajabi F., Ranjbar F. (2016). Desalin. Water Treat..

[cit18] Kluczka J., Trojanowska J., Zołotajkin M. (2015). Desalin. Water Treat..

[cit19] Kıpcak I., Özdemir M. (2012). Chem. Eng. J..

[cit20] Cengeloglu Y., Tor A., Arslan G., Ersoz M., Gezgin S. (2007). J. Hazard. Mater..

[cit21] Li X., Liu R., Wu S., Liu J., Cai S., Chen D. (2011). J. Colloid Interface Sci..

[cit22] Tural S., Ece M. Ş., Tural B. (2018). Ecotoxicol. Environ. Saf..

[cit23] Ting T. M., Nasef M. M., Hashim K. (2016). J. Chem. Technol. Biotechnol..

[cit24] Santos P. V. S., Libânio M., Teixeira M. C. (2024). Sci. Total Environ..

[cit25] Kurczewska J., Stachowiak M., Cegłowski M. (2024). Environ. Res..

[cit26] Zhuang S., Zhu K., Xu L., Hu J., Wang J. (2022). Sci. Total Environ..

[cit27] Luo M., Zhu C., Chen Q., Song F., Hao W., Shen Z., Konhauser K. O., Alessi D. S., Zhong C. (2023). Colloids Surf., A.

[cit28] Azlan K., Wan Saime W. N., Lai Ken L. (2009). J. Environ. Sci..

[cit29] de Farias B. S., Sant'Anna Cadaval Jr T. R., de Almeida Pinto L. A. (2019). Int. J. Biol. Macromol..

[cit30] Ho H. Q. , Synthesis of eco-friendly adsorbents for the removal of contaminants in wastewater, Tokushima University, 2019, https://ci.nii.ac.jp/naid/500001352478

[cit31] Kavitha K., Bharathi S., Rajalakshmi A., Ramesh M., Padmini P., Suresh G., Guruchandran V., Prakash M., Puvanakrishnan R., Ramesh B. (2024). Part. Sci. Technol..

[cit32] Raik S. V., Gasilova E. R., Dubashynskaya N. V., Dobrodumov A. V., Skorik Y. A. (2020). Int. J. Biol. Macromol..

[cit33] de Farias B. S., Sant'Anna Cadaval Junior T. R., de Almeida Pinto L. A. (2019). Int. J. Biol. Macromol..

[cit34] Zhao B., Lou C., Zhou Q., Zhu Y., Li W., Jingshan M. (2022). RSC Adv..

[cit35] Bian Y., Gao D., Liu Y., Li N., Zhang X., Zheng R. Y., Wang Q., Luo L., Dai K. (2015). RSC Adv..

[cit36] Schott J., Kretzschmar J., Acker M., Eidner S., Kumke M. U., Drobot B., Barkleit A., Taut S., Brendler V., Stumpf T. (2014). Dalton Trans..

[cit37] Zhang X., Wang J., Chen S., Bao Z., Xing H., Zhang Z., Su B., Yang Q., Yang Y., Ren Q. (2017). Sep. Purif. Technol..

[cit38] Putro J. N., Kurniawan A., Ismadji S., Ju Y.-H. (2017). Environ. Nanotechnol., Monit. Manage..

[cit39] Nanta P., Kasemwong K., Skolpap W. (2018). J. Environ. Chem. Eng..

[cit40] Kavak D. (2009). J. Hazard. Mater..

[cit41] Che Man H., Chin W. H., Zadeh M. R., Yusof M. R. M. (2012). BioResources.

[cit42] Wang B., Lin H., Guo X., Bai P. (2014). Desalination.

[cit43] Kluczka J., Gnus M., Dudek G., Turczyn R. (2017). Sep. Sci. Technol..

[cit44] Zhu H. Y., Jiang R., Fu Y. Q., Jiang J. H., Xiao L., Zeng G. M. (2011). Appl. Surf. Sci..

[cit45] Chang G., Bao Z., Zhang Z., Xing H., Su B., Yang Y., Ren Q. (2014). Ind. Eng. Chem. Res..

[cit46] Chang G., Bao Z., Zhang Z., Xing H., Su B., Yang Y., Ren Q. (2013). J. Colloid Interface Sci..

[cit47] Zhang W., Oswal H., Renew J., Ellison K., Huang C.-H. (2019). J. Hazard. Mater..

[cit48] Huang Y. H., Peddi P. K., Tang C., Zeng H., Teng X. (2013). Sep. Purif. Technol..

[cit49] Zhang W., Oswal H., Renew J. E., Gallagher B., Ellison K., Huang C.-H. (2020). J. Hazard. Mater..

