# Supplementary material for: Hydroxyl-modified chitosan nanofiber beads for sustainable boron removal and environmental applications
Source: RSC Adv. 2025 Mar 4;15(9):7090–102. doi: 10.1039/d5ra00077g (PMC11878523; doi:10.1039/d5ra00077g)
Supplement: RA-015-D5RA00077G-s001 [file RA-015-D5RA00077G-s001.zip › supporting_information1.docx]

Supporting Information for

**Hydroxyl-Modified Chitosan Nanofiber Beads for Sustainable Boron Removal and Environmental Applications**

Ho Hong Quyen,*^a^ Hoang M.Nguyen,*^a^ Vu Chi Mai Tran,^a^ Phuoc-Cuong Le,^a^ Masashi Kurashina,^b^ Mikito Yasuzawa,^b^ and Yuki Hiraga^c^

^a^ The University of Da Nang - University of Science and Technology, 54 Nguyen Luong Bang Street, Lien Chieu District, Da Nang City 550000, Vietnam.

^b^ Department of Applied Chemistry, Graduate School of Science and Technology, Tokushima University, 2-1 Minamijosanjima-cho, Tokushima-shi, Tokushima 770-8506, Japan.

^c^ Department of Chemical and Biological Technologies, Shikoku Research Institute, Inc., 2109

Yashima-Nishimachi, Takamatsu-shi, Kagawa 761-0192, Japan.

^*^Corresponding authors:

Email addresses:

[hhquyen@dut.udn.vn](mailto:hhquyen@dut.udn.vn) (Dr. Ho Hong Quyen).

[nmhoang@dut.udn.vn](mailto:nmhoang@dut.udn.vn) (Dr. Hoang M. Nguyen).


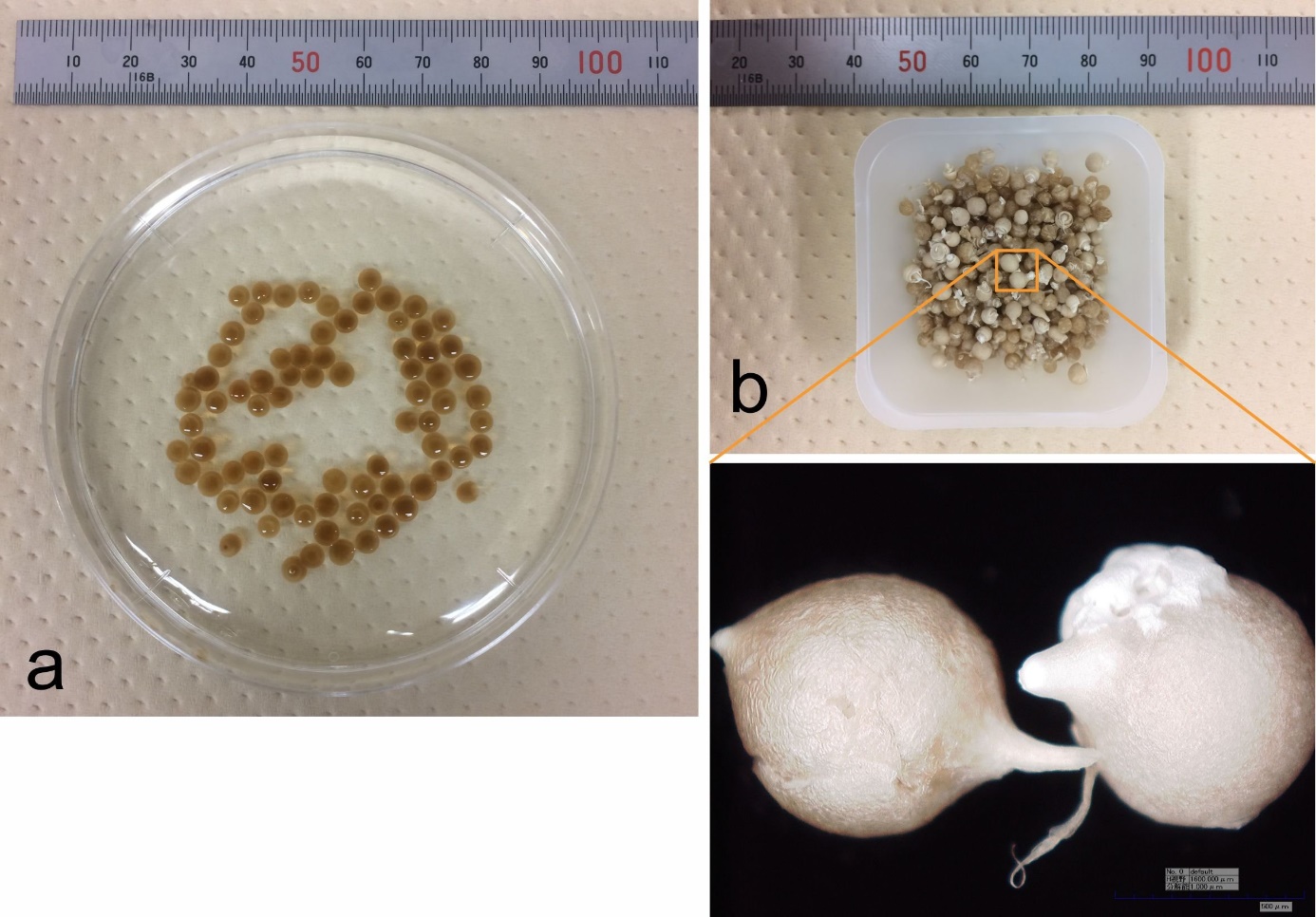


**Fig. S1.** Illustration of CGCNF beads (a) before and (b) after freeze-drying.

| 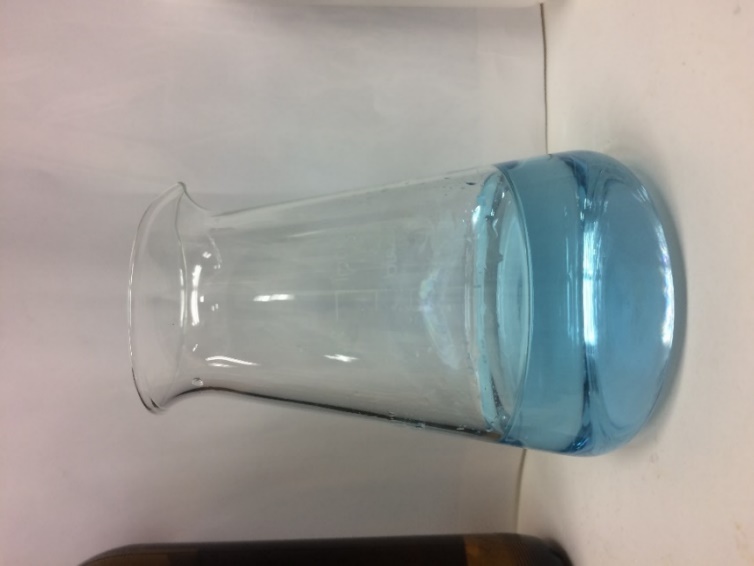  a  b | 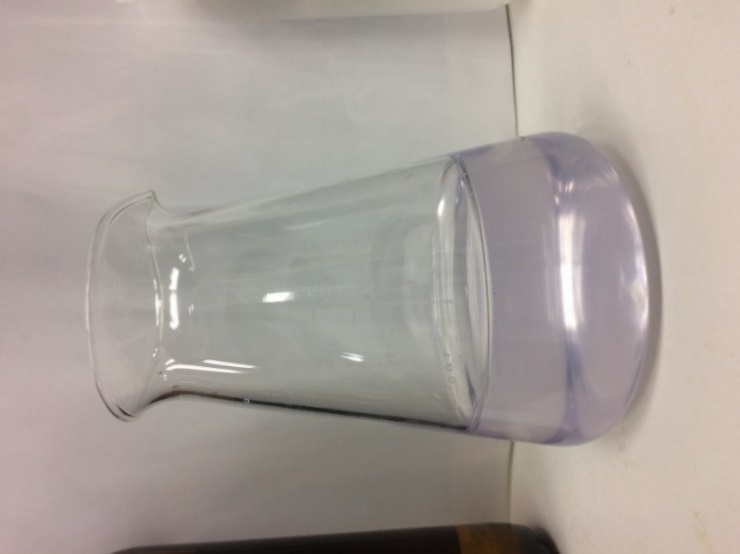 |
| --- | --- |

**Fig. S2.** Experiment of colloidal titration (a) before and (b) after adding indicator of toluidine blue.


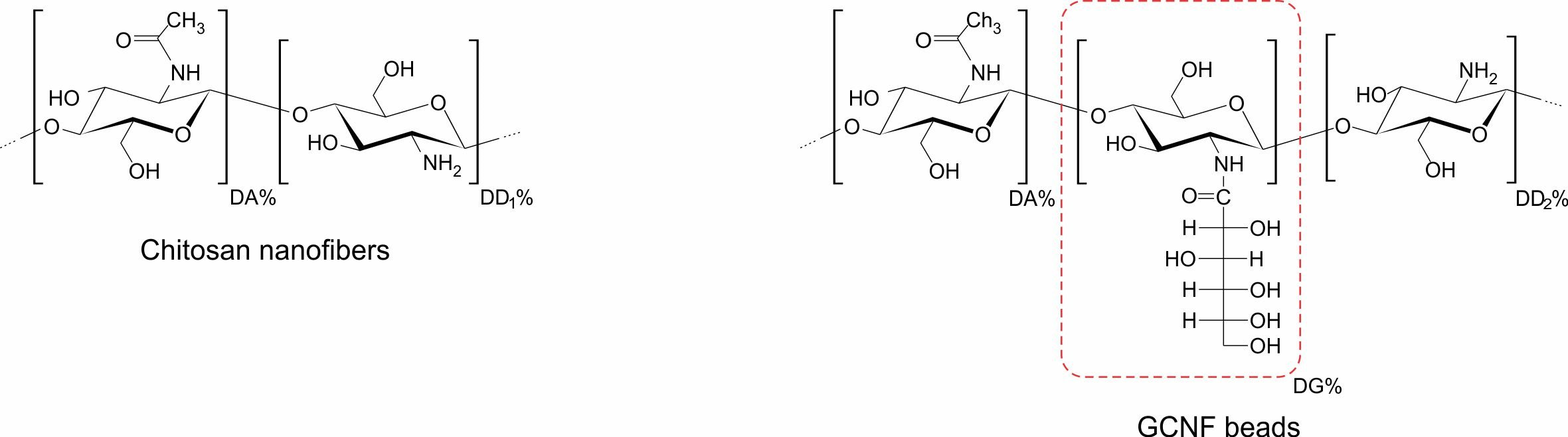


**Fig. S3.** *DA*%, *DD*%, and *DG*% of chitosan nanofibers and GCNF beads.

**Calculation details of degree of deacetylation (*DD*)**

*DD_1_*% of bare chitosan nanofibers was calculated according to **Eq. (S1)**:

${DD}_{1}\left( \text{\%} \right)\text{ = }\frac{d_{1}}{\frac{W_{1}\text{ }\text{- 161}d_{1}}{\text{204}}\text{ + }d_{1}}\text{100 }$ (S1)

Where *d*_1_ is the mole of deacetylated unit (mol), *W*_1_ is the dry mass of bare chitosan nanofibers (g), and 161 and 204 are the molar weight of deacetylated unit and acetylated unit (g/mol), respectively.

*DD_2_*% of GC particles and GCNF beads was calculated based on **Eq. (S2):**

${DD}_{2}\left( \text{\%} \right)\text{ = }\frac{d_{2}}{\frac{W_{2}\text{ }\text{- 204}{a-161d}_{1}}{\text{340}}\text{ +}\text{a+}d_{2}}\text{100 }$ (S2)

Where *d*_2_ is the mole of deacetylated unit (mol), *a* is the mole of acetylated unit (mol), 340 is the molar weight of gluconated unit (g/mol), and *W*_2_ is the dry mass of GC particles or GCNF beads (g).

The degree of gluconated units (*DG*%) is therefore given as follows:

*DG*(%) = *DD*_1_(%) – *DD*_2_(%) (S3)

The level of grafted gluconted units (*LGG*%) is written as follows:

$LGG\left( \text{\%} \right)\text{ = }\frac{DG}{{DD}_{1}}\text{100 }$ (S4)

**Table S1.** The solubility of various adsorbents.

| **Sample** | **Mili-Q water** | **0.1M NaOH** | **6M NaOH** | **0.1M HCl** | **6M HCl** |
| --- | --- | --- | --- | --- | --- |
| GC particles | + | + | + | - | - |
| GCNF beads | + | + | + | - | - |
| CGC particles | + | + | + | + | + |
| CGCNF beads | + | + | + | + | + |

“+”: insoluble.

“-”: soluble.


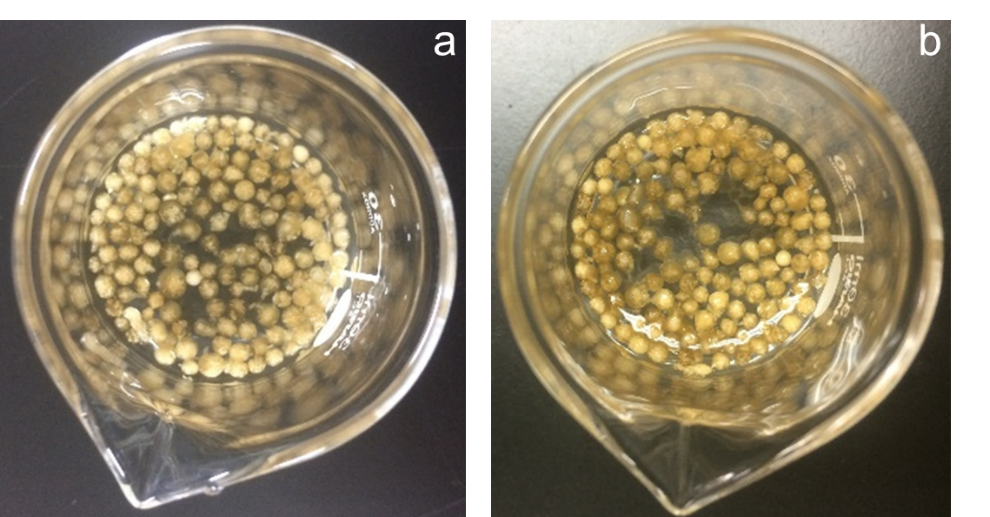


**Fig. S4.** CGCNF beads in 6M HCl solution after (**a**) 1 min; and (**b**) 1 month.


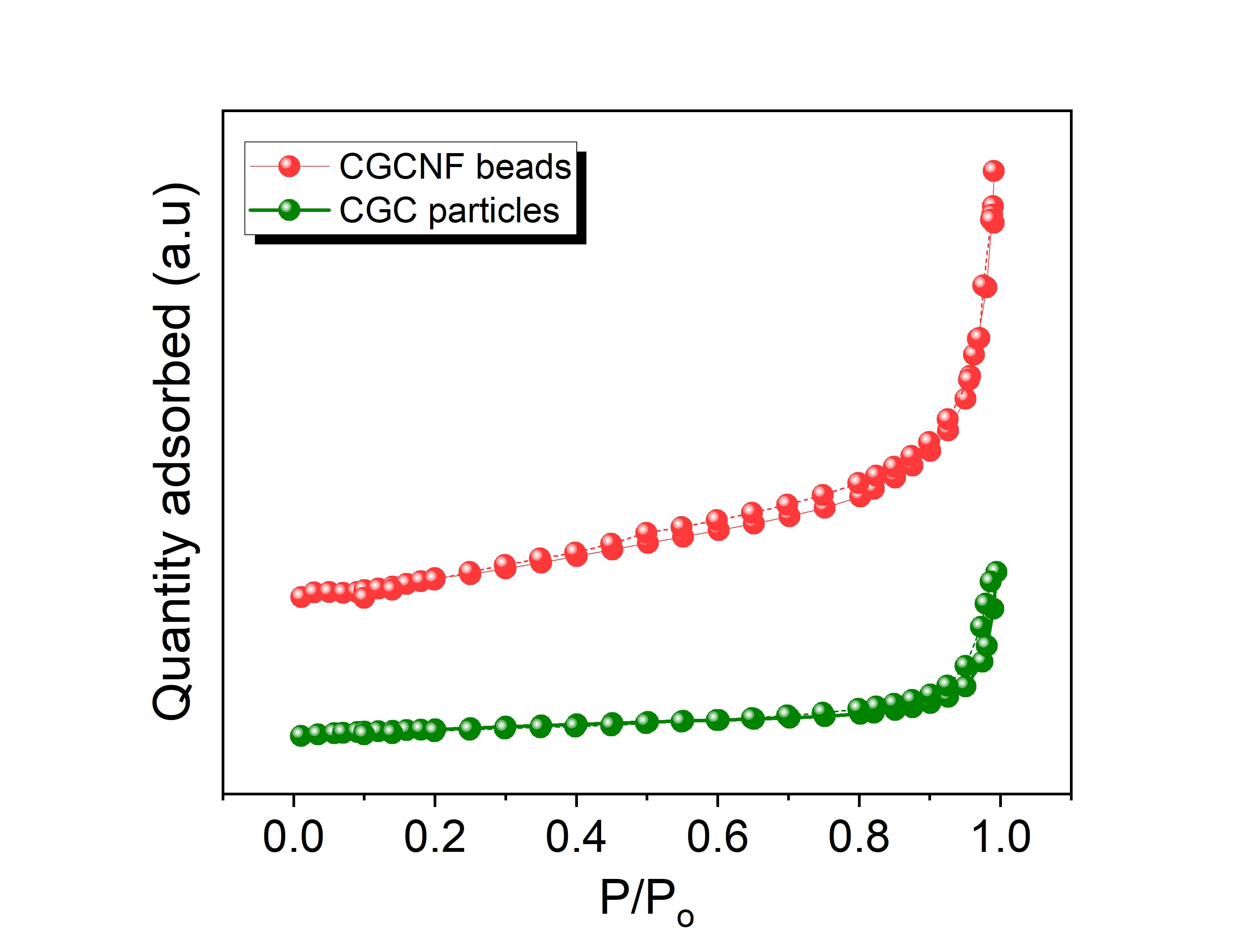


**Fig. S5.** N_2_ adsorption-desorption isotherm curves of CGCNF beads and CGC particles samples.


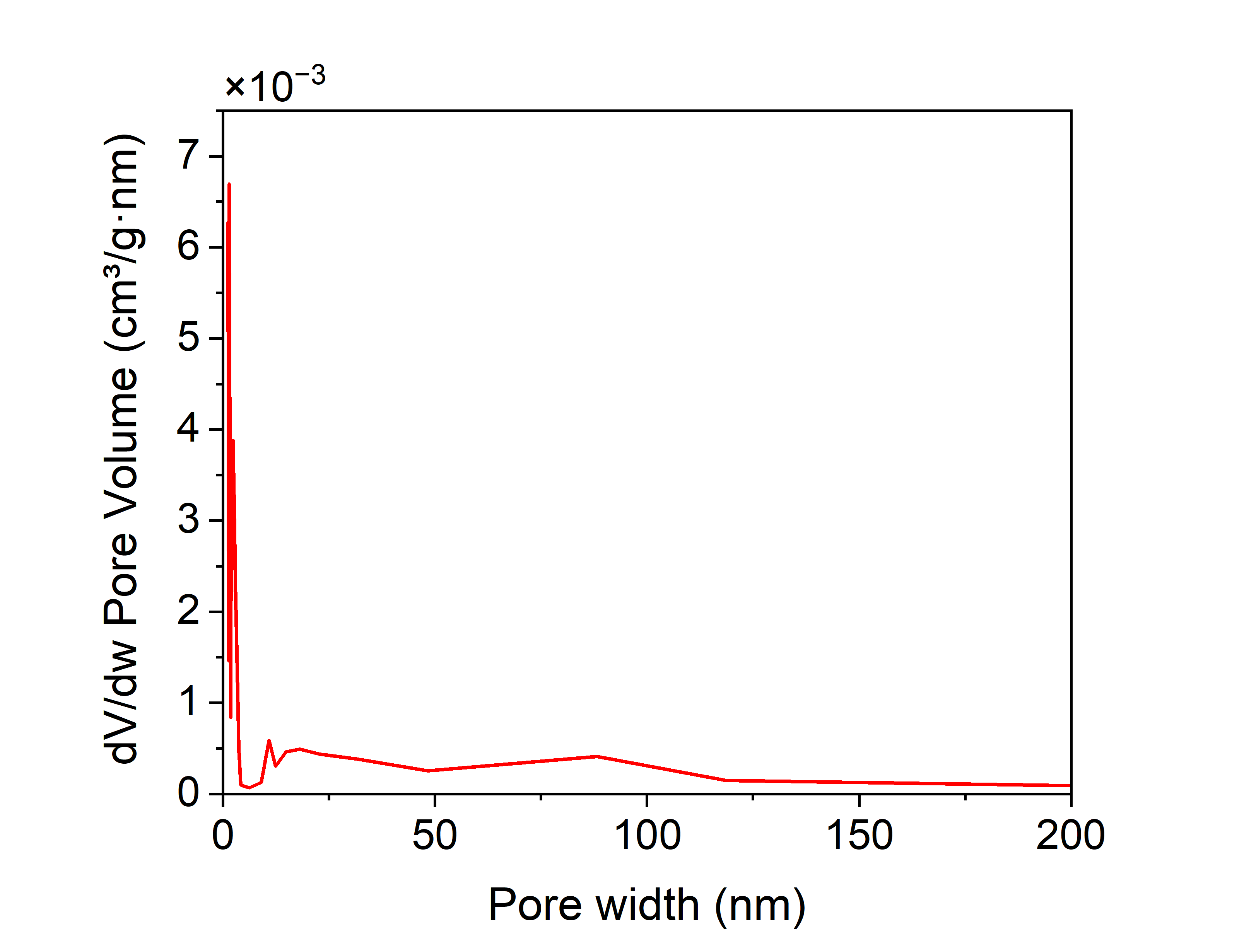


**Fig. S6.** Pore size distribution of CGCNF sample.

**
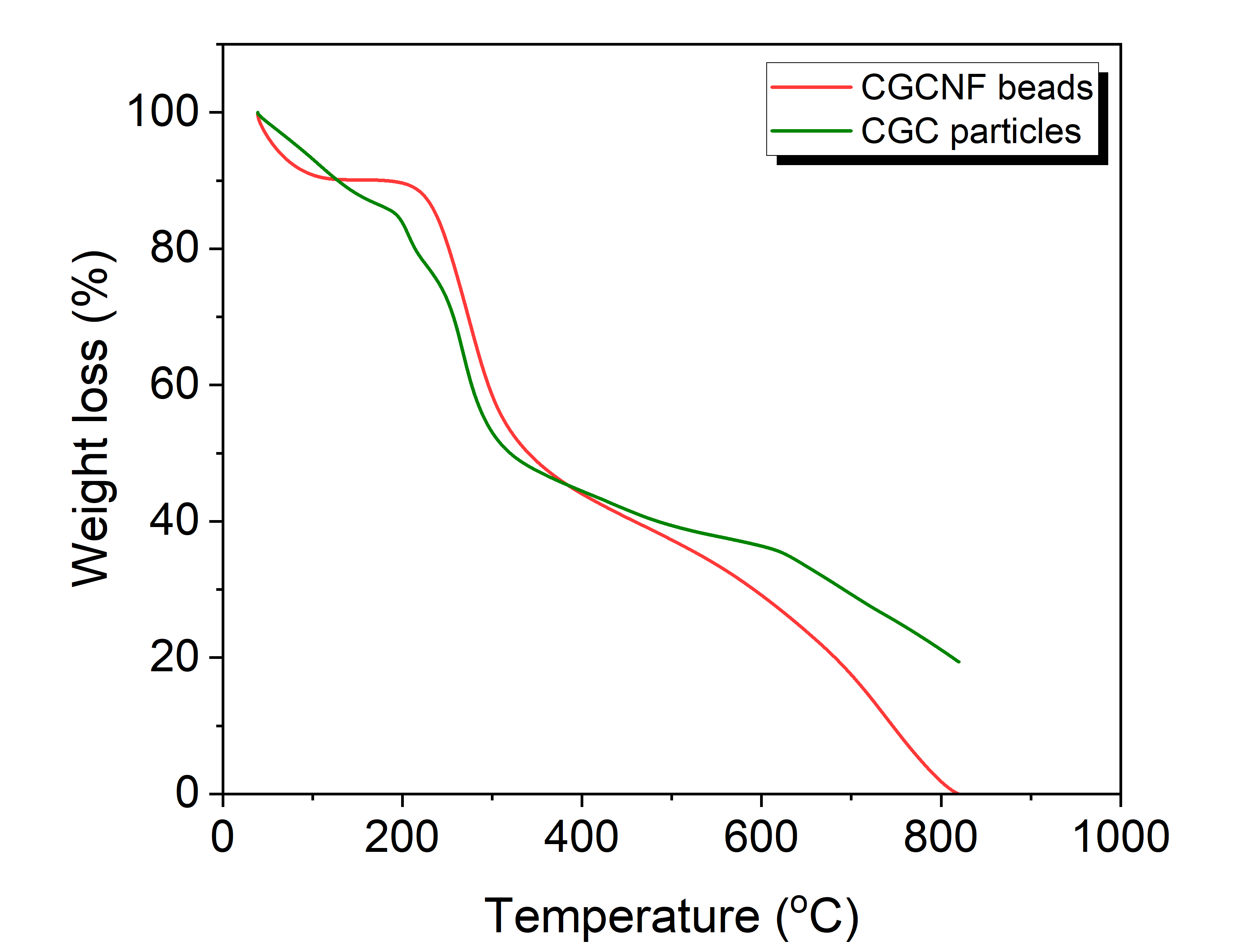
**

**Fig. S7.** TGA analysis for the prepared chitosan-based samples.

**Fig. S8.** Effect of initial pH solution on boron removal efficiency by using CGCNF beads and CGC particles (Initial concentration of boron: 400 mg/L, mass of adsorbent: 0.8 g, initial pH solution: 2.03-12.03, solution volume: 20 mL, contact time: 24 h and temperature: 25 ºC).

**Table S2.** Langmuir, Freundlich, and Temkin isotherm constants and correlation coefficients of various adsorbents for boron adsorption.

| Isotherm | Parameters | CGCNF beads | CGC particles | Amberlite IRA-743 |
| --- | --- | --- | --- | --- |
| Langmuir | *q*_exp_ (mg/g) | 5.53 | 3.96 | 5.42 |
|  | *q*_max_ (mg/g) | 6.05 | 4.13 | 5.73 |
|  | *b* (L/mg) | 0.094 | 0.090 | 0.151 |
|  | *R*^2^ | 0.9976 | 0.9991 | 0.9997 |
|  | *R*_L_ range | 0.027 - 0.513 | 0.028 - 0.524 | 0.016 - 0.385 |
| Freundlich | *K*_F_ | 1.793 | 0.886 | 1.827 |
|  | *n* | 4.454 | 3.441 | 4.522 |
|  | *R*^2^ | 0.9441 | 0.8418 | 0.7764 |
| Temkin | *B* (J/mol) | 0.589 | 0.716 | 0.963 |
|  | *A*_T_ (L/g) | 44.003 | 1.478 | 2.617 |
|  | *R*^2^ | 0.9687 | 0.9866 | 0.9111 |

c

**Fig. S9.** Experimental data of boron adsorption onto CGCNF beads fitted to linearized forms of **(a)** Langmuir, **(b)** Freundlich, and **(c)** Temkin isotherms (Initial concentration of boron: 10 - 400 mg/L, mass of adsorbent: 0.8 g, initial pH solution: 5.45, solution volume: 20 mL and contact time: 24h).

c

**Fig. S10.** Experimental data of boron adsorption onto CGC particles fitted to linearized forms of **(a)** Langmuir, **(b)** Freundlich, and **(c)** Temkin isotherms (Initial concentration of boron: 10 - 400 mg/L, mass of adsorbent: 0.8 g, initial pH solution: 5.45, solution volume: 20 mL and contact time: 24 h).

**Table S3.** Kinetics parameters for adsorption of boron by using various adsorbents.

| **Kinetic models** | **Parameters** | **CGCNF beads** | **CGC particles** |
| --- | --- | --- | --- |
| Pseudo-first order model | *k*_1_ (min^-1^) | 0.0648 | 0.0037 |
|  | *q*_e_ (mg/g) | 5.47 | 4.06 |
|  | *R*^2^ | 0.9958 | 0.9137 |
| Pseudo-second order | *k*_2_ (g mg^-1^ min^-1^) | 0.0221 | 0.0007 |
|  | *q*_e_ (mg/g) | 5.67 | 5.04 |
|  | *R*^2^ | 1.000 | 0.9901 |
| Intra-particle diffusion | *K*_diff_ (mg g^-1^ min^-1/2^) | 0.0370 | 0.1231 |
|  | *C* (mg g^-1^) | 4.5003 | 0.0359 |
|  | *R*^2^ | 0.3860 | 0.9137 |

a

**Fig. S11.** Experimental data of boron adsorption onto CGCNF beads fitted to linearized forms of **(a)** Pseudo-first order, **(b)** Pseudo-second order, and **(c)** Intra-particle diffusion (Initial concentration of boron: 400 mg/L, mass of adsorbent: 0.8 g, initial pH solution: 5.56, solution volume: 20 mL, contact time: 0-24 h and temperature: 25 ^o^C).

**Fig. S12.** Experimental data of boron adsorption onto CGC particles fitted to linearized forms of **(a)** Pseudo-first order, **(b)** Pseudo-second order, and **(c)** Intra-particle diffusion (Initial concentration of boron: 400 mg/L, mass of adsorbent: 0.8 g, initial pH solution: 5.56, solution volume: 20 mL, contact time: 0-24 h and temperature: 25 ^o^C).


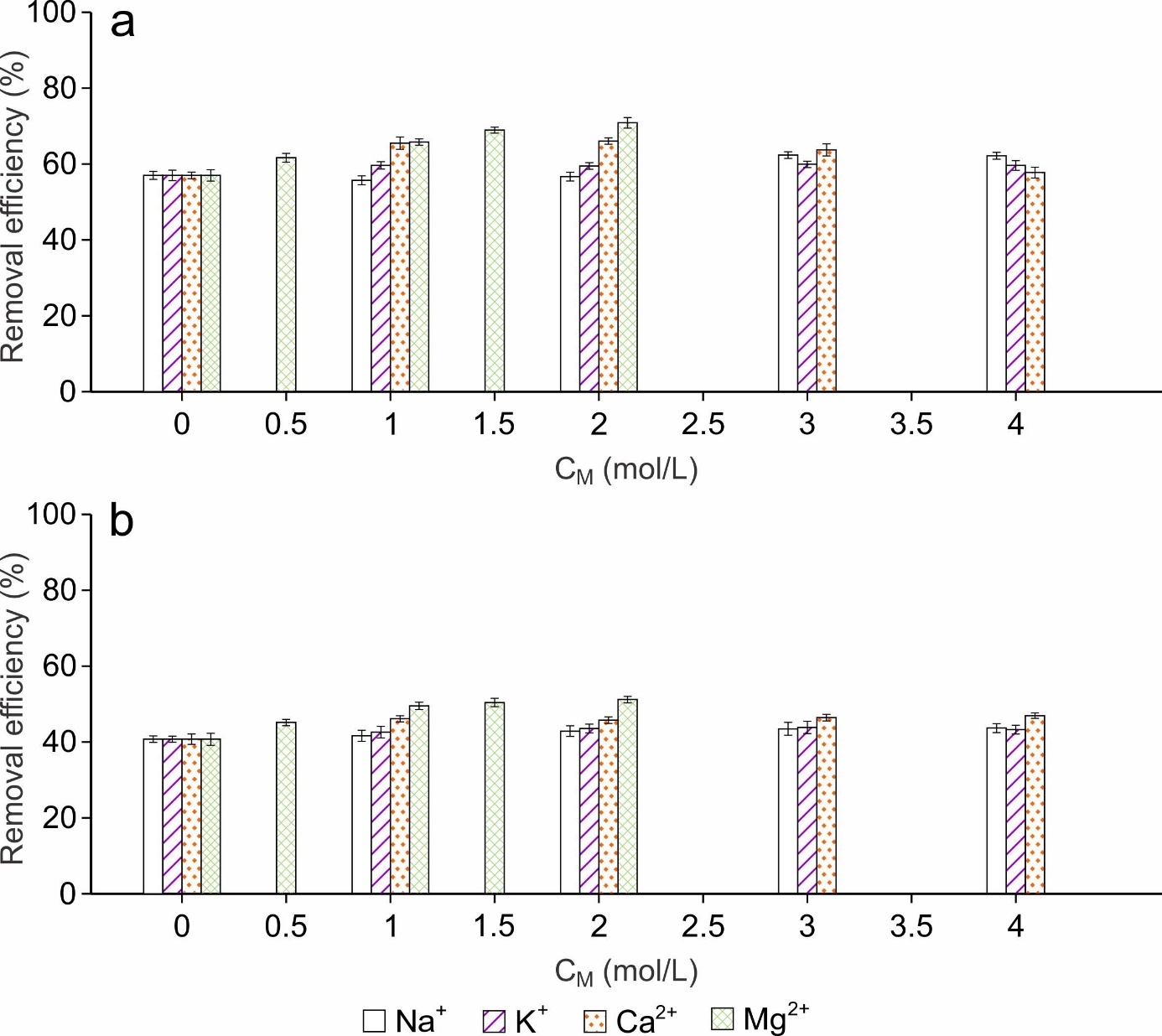


**Fig. S13.** Effect of ions Na^+^, K^+^, Ca^2+^ and Mg^2+^ on boron removal efficiency by using (a) CGCNF beads and (b) CGC particles.

**Fig. S14.** Adsorption performance of Se(VI), As(III), As(V), Cr(III) and Cr(VI) on CGCNF beads and CGC particles.


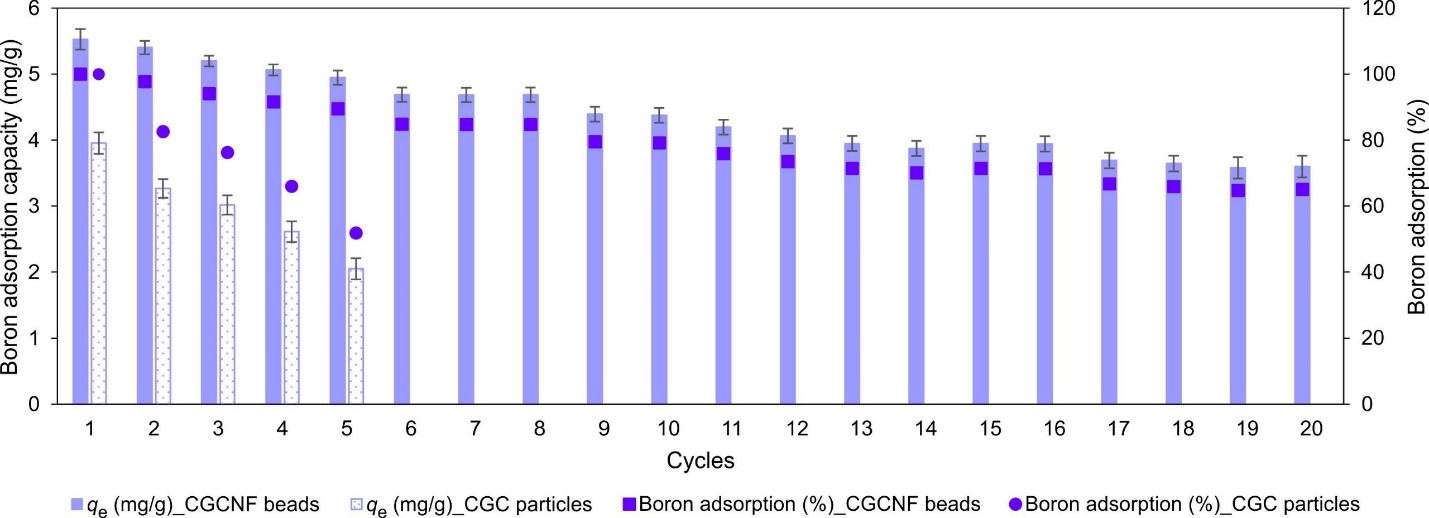


**Fig. S15.** Desorption and reusability of CGCNF beads and CGC particles (Initial concentration of boron: 400 mg/L, mass of adsorbent: 0.8 g, initial pH solution: 5.56, solution volume: 20 mL, contact time: 24h and temperature: 25 ^o^C).

**Table S4.** Comparison of boron desorption efficiency by various adsorbents.

| **Adsorbent** | **Desorption condition** | **Regeneration condition** | **Desorption efficiency** | **Ref.** |
| --- | --- | --- | --- | --- |
| Functional magnetic mesoporous silica hybrid nanoparticles | *C*_i_ = 100 mg/L, dose = 0.1 g/50 mL, 0.1 M HCl, 12h, room temperature | 3% NH_3_.H_2_O then dried at 60^o^C | 65% after 7 cycles | [51] |
| Magnetic multi-hydroxyl  microbeads | *C*_i_ = 150 mg/L, m = 0.1 g, 0.1 M HCl, 8h, 25 ^o^C | Water at pH 9 then dried at 80 ^o^C | 44.5% after 6 cycles | [51] |
| Zeolitic imidazolate framework | dose = 0.1 g/80 mL, water, 12h | 80 ^o^C, overnight | 74.4% after 4 cycles | [52] |
| CGC particles | *C*_i_ = 400 mg/L, dose = 0.8 g/50 mL, 0.1 M HCl, 24h, 25 ^o^C | 0.1 M NaOH, 12 h, 25^o^C then freeze drying | 51.8% after 5 cycles | This work |
| CGCNF beads | *C*_i_ = 400 mg/L, dose = 0.8 g/50 mL, 0.1 M HCl, 24h, 25 ^o^C | 0.1 M NaOH, 12 h, 25^o^C then freeze drying | 65.1% after 20 cycles | This work |

**Table S5.** Characteristics of FGD wastewater (concentrations in mg/L).

| **Parameters** | **FGD wastewater** |
| --- | --- |
| pH | 6.6 |
| Na^+^ | 1261.40 |
| Ca^2+^ | 3627.85 |
| Mg^2+^ | 1392.50 |
| B^3+^ | 133.29 |
| Se^6+^ | 850.38 |
| As^3+^ | 0.02 |
| Cr^5+^ | 0.04 |
| Zn^2+^ | 1.49 |
| Cu^2+^ | 0.44 |
| Cd^2+^ | 0.03 |
| Ni^2+^ | 0.15 |
